# A review of deep learning architectures for plant disease detection

**DOI:** 10.55730/1300-0152.2761

**Published:** 2025-09-09

**Authors:** Yasin KAYA, Ercan GÜRSOY

**Affiliations:** 1 Department of Artificial Intelligence Engineering, Faculty of Computer and Informatics, Adana Alparslan Turkes Science and Technology University, Adana, Turkiye; 2 Department of Computer Engineering, Faculty of Computer and Informatics, Adana Alparslan Turkes Science and Technology University, Adana, Turkiye

**Keywords:** Plant disease, disease detection, deep learning, CNN

## Abstract

**Background/aim:**

The rapid advancement of deep learning (DL) has revolutionized plant disease detection by enabling highly accurate, image-based diagnostic solutions. This review provides a comprehensive synthesis of DL-based methodologies for plant disease detection, systematically structured around the key stages of the modeling pipeline, encompassing data acquisition, preprocessing, augmentation, classification, detection, segmentation, and deployment.

**Materials and methods:**

The review focuses on evaluating convolutional neural network (CNN) architectures such as VGG, ResNet, EfficientNet, and DenseNet across diverse experimental contexts. Classification strategies are categorized according to their integration of visualization techniques (e.g., saliency maps, Grad-CAM) to enhance model interpretability, emphasizing the pivotal role of explainable artificial intelligence (XAI) in plant pathology. Object detection models are systematically examined within both one-stage (YOLO, SSD) and two-stage (Faster R-CNN) paradigms. Furthermore, critical challenges—such as environmental variability, data imbalance, and computational constraints—along with potential solutions including transfer learning, synthetic data generation using generative adversarial networks (GANs) and diffusion models, and edge computing for real-time deployment, are comprehensively discussed.

**Results:**

This review summarizes best practices for dataset selection and model optimization for mobile platforms, emphasizing their role in improving the efficiency and accuracy of plant disease detection systems.

**Conclusion:**

Deep learning-based methods show strong potential to enhance precision and resilience in real-world plant disease detection and monitoring.

## 1. Introduction

Population growth has driven a steady increase in the demand for various plant products. Various crop protection strategies have been employed for decades to combat plant diseases, playing a crucial role in meeting the increasing demand for large quantities of plant-based food products (Kaya and Gursoy, 2023). However, the rising costs associated with pest infestations and plant diseases pose an increasingly serious threat to crop yields, thereby jeopardizing harvest success. According to the Food and Agriculture Organization (FAO), plant diseases cause approximately 20%–40% of global crop losses each year, posing a major threat to food security and sustainable agriculture (FAO, 2021). Furthermore, biotic stresses represent a major threat to agricultural yields, leading to considerable losses in food production. Plants play vital roles in society by providing economic and social benefits, supporting environmental protection, advancing agricultural development, and ensuring food supply. However, plant-related disciplines, including species identification, early disease detection, and yield estimation, often require specialized expertise and considerable human effort (Kaya and Gursoy, 2023; Cetinkaya and Tandirovic Gursel, 2025).

Plant diseases represent a major challenge in agriculture, exerting a substantial impact on crop production. They contribute substantially to reduced crop yields and adversely affect both the operational efficiency and financial performance of farms. They also reduce productivity and pose significant obstacles to the smooth operation of farming activities. The impact of plant diseases is extensive, encompassing issues from farm-level productivity losses to global food safety concerns. Technical expertise and effective management of plant diseases are therefore essential for achieving sustainable agriculture.

Plant diseases are primarily caused by diverse bacteria, fungi, viruses, and other naturally occurring pathogens throughout the plant life cycle. Several methods have been developed for identifying and classifying different leaf diseases. The most common technique is direct visual inspection with the naked eye; however, it is not always sufficiently effective. This approach can be time-consuming and demands prompt decision-making to prevent the spread of disease to healthy plants. Owing to these limitations, researchers increasingly rely on machine learning (ML) and deep learning (DL) methods for plant disease detection and classification. Plant disease detection has emerged as a commercially viable research domain integrating image processing, ML, DL, and computer vision techniques. These advanced technologies facilitate efficient and timely disease detection, resulting in healthier plants and improved crop yields.

### 1.1. Challenges

#### 1.1.1. Environmental factors

Environmental factors play a critical role in the onset and spread of plant diseases. Disease symptoms are influenced by temperature, humidity, and soil conditions, which complicates the development of reliable detection models. Climate change profoundly affects various stages of a plant’s life cycle, with temperature fluctuations influencing key phenological events such as flowering and leaf emergence (Keller and Shea, 2021). Changes in these ecological variables can disrupt species synchrony, resulting in ecological imbalance.

To address these challenges, Isinkaye et al. (2024) proposed a novel approach combining DL with content-based filtering to enhance plant disease detection and treatment recommendations. Their study highlights the influence of environmental conditions on the variability of disease symptoms and detection accuracy. Similarly, Corlouer et al. (2024) introduced the concept of ecological classification, integrating environmental data with quantitative genetics to improve the performance of plant disease detection models.

In addition, Lee and Yun (2023) developed a model incorporating historical environmental data—including temperature, humidity, and soil parameters—to more accurately assess plant disease risk. Their results emphasize the importance of incorporating environmental parameters into disease prediction models to enhance model robustness and practical applicability.

#### 1.1.2. Symptom variability

The variability of plant disease symptoms presents a major challenge for DL-based plant disease detection. The same plant disease may manifest differently depending on various environmental and biological factors. Several strategies have been proposed to address symptom variability. A large dataset containing images of diseased plants exhibiting diverse symptoms is employed as one approach in plant disease detection studies. This enables the model to recognize multiple types of plant diseases more effectively (Ferentinos, 2018).

Another approach involves the use of a technique known as transfer learning. In transfer learning, a model is pretrained on a large dataset of images unrelated to the target domain, such as natural or medical images (Dinc and Kaya, 2024; Tandirovic Gursel and Kaya, 2025). The model is subsequently fine-tuned using a smaller dataset of diseased plant images. This approach helps the model learn generalizable features relevant to plant disease detection and enhances its robustness to symptom variability (Brahimi et al., 2018; Arnal Barbedo, 2019).

Researchers have also developed novel DL architectures specifically designed for plant disease identification. These architectures are designed to learn complex relationships among multiple features within an image. This enables more accurate identification of plant diseases, even when symptom expressions vary (Lu et al., 2022). These studies highlight the importance of employing large datasets, transfer learning, and advanced DL architectures to improve the reliability of plant disease detection models despite symptom variability.

#### 1.1.3. Image processing challenges

Image processing is a fundamental component of plant disease detection; however, several challenges limit its efficiency. These challenges include image noise, blurring, inconsistent data acquisition conditions, and the inherent complexity of feature extraction.

Lighting variability and limited imaging equipment commonly contribute to noise and blurring, which can obscure critical visual details required for accurate disease identification. Deep architectures such as deep belief networks and deep Boltzmann machines have been developed to address these challenges by effectively denoising images and extracting robust features to ensure high classification accuracy for diseased plant images (Shoaib et al., 2023).

Collecting high-quality agricultural images poses several challenges, including variable weather conditions, uneven illumination, and diverse camera perspectives. These factors contribute to image quality variability, complicating the accuracy of disease detection. The use of unmanned aerial vehicles (UAVs) for image acquisition introduces additional limitations, including weather dependency and restricted flight duration (Neupane and Baysal-Gurel, 2021).

Filtering techniques such as box, Gaussian, gradient, and Laplacian filters are employed to mitigate noise-related issues. These filters enhance image quality by smoothing noise while preserving critical structural features. They are often combined with deep denoising autoencoders (DDA) to remove noise from plant leaf images and improve the accuracy of subsequent disease detection models (Shoaib et al., 2023).

Extracting discriminative features from plant images is challenging due to the diverse visual manifestations of diseases and complex natural backgrounds. Advanced feature extraction techniques such as thresholding, edge detection, template matching, Hough transform, and histogram of oriented gradients (HoG) are used to identify diseased regions. The irregular occurrence of diseases and heterogeneity of symptoms necessitate efficient feature extraction techniques (Sarkar et al., 2023).

### 1.2. Objectives

This study aims to provide a comprehensive overview of research on plant disease detection and to explain the various methodological approaches employed by researchers in the literature. The review covers several key stages, including image preprocessing, segmentation, feature extraction, and classification. A comprehensive analysis of practical DL algorithms applied to plant disease diagnosis is also presented. This review specifically focuses on studies conducted between 2018 and 2023. To illustrate the quality of research during this period, we present a list of SCI- and Scopus-indexed publications along with their key contributions. This process provides an overview of the current literature and serves as a practical guide for newcomers to the field of plant disease detection. The review is intended as a reference tool to facilitate clear understanding of the methods and models used in this research domain.

### 1.3. Contributions

This paper presents an overview of plant disease detection methods developed between 2018 and 2024. To facilitate the work of researchers, the key aspects are summarized as follows:

- The review outlines the advantages and limitations of employing DL methods for plant disease detection.
- Particular emphasis is placed on algorithms commonly applied in this context to distinguish between ML and DL models.
- A comprehensive set of tables lists all datasets used in the reviewed studies and provides relevant details for each.
- The overview illustrates the distribution of selected studies across multiple sectors, including universities, governmental institutions, private organizations, industries, and academia–industry collaborations.
- The review assists researchers in selecting appropriate methods and datasets according to their research objectives. This review serves as a valuable resource for researchers seeking an overview of leaf disease detection methods conducted within the specified timeframe.

### 1.4. Search process

To ensure a comprehensive and systematic literature search on DL-based plant disease detection, a well-defined search process is essential. This process involves several essential steps to collect relevant scientific articles and datasets.

#### 1.4.1. Database selection

To identify relevant literature, several well-established academic databases were utilized, including:

- **IEEE Xplore**—for artificial intelligence (AI) and computer vision applications in agriculture.
- **ScienceDirect**—for research in agricultural and biological sciences.
- **SpringerLink—**for multidisciplinary studies involving deep learning applications.
- **Google Scholar**—for a broad range of related scholarly articles.
- **PubMed**—for bioinformatics-related research on plant diseases.

#### 1.4.2. Search keywords and query design

Search queries were systematically formulated to retrieve high-quality and peer-reviewed publications. The following keywords and Boolean operators were employed:

- (“Deep Learning” OR “Convolutional Neural Network” OR “Machine Learning”) AND (“Plant Disease” OR “Leaf Disease”) AND (“Detection” OR “Classification”)
- (“Image Processing” AND “Plant Health Monitoring”)
- (“Hyperspectral Imaging” OR “Multispectral Imaging”) AND (“Disease Diagnosis”)

#### 1.4.3. Inclusion and exclusion criteria

To ensure the relevance and quality of the selected studies, specific inclusion and exclusion criteria were applied.

##### Inclusion

- Peer-reviewed journal articles.
- Studies published from 2018 onwards.
- Experimental studies focusing on real-world plant disease detection.
- Articles providing open-source or locally collected datasets.

##### Exclusion

- Studies lacking experimental validation.
- Studies employing outdated methodologies.
- Review papers that do not provide novel insights or findings.
- Preprints hosted on servers such as arXiv and bioRxiv were excluded.

#### 1.4.4. Data extraction and categorization

The extracted studies were categorized according to the following criteria:

- **Deep learning architecture used—**for example, convolutional neural networks (CNNs), recurrent neural networks (RNNs), and transformer-based models.
- **Type of dataset**—including PlantVillage, real-world agricultural field images, and synthetic datasets.
- **Evaluation metrics**—such as accuracy, precision, recall, F1-score, AUC-ROC, and mean average precision (mAP).

#### 1.4.5. Verification and cross-referencing

Cross-referencing was employed to verify citations in highly cited studies, ensuring that no significant work was overlooked. This systematic search process ensures a comprehensive and up-to-date literature review, establishing a robust foundation for analyzing recent advances in DL-based plant disease detection.

## 2. Deep learning

The concept of DL was first introduced by Hinton et al. (2006). However, the foundation of deep neural networks (DNNs) was established much earlier, with notable contributions from Lecun et al. (1998) on convolutional neural networks (CNNs) and Goodfellow et al. (2016) on the general principles of deep learning (DL). DL utilizes DNNs for data analysis and hierarchical feature learning. Each layer functions as a processing unit, extracting low-level features and integrating them into higher-level representations. This hierarchical feature extraction enables DL models to automatically learn complex patterns from raw data. This approach overcomes the limitations of conventional algorithms that depend heavily on manually engineered features.

Traditional image classification and recognition methods often rely on manually designed features (Topuz and Kaya, 2025), limiting their ability to identify complex patterns. DL overcomes these limitations through unsupervised learning directly from raw images, enabling the extraction of multilevel features that capture low-, mid-, and high-level semantic information (Kamilaris and Prenafeta-Boldú, 2018; Hamroun et al., 2025; Kaya et al., 2025). This capability makes DL a robust framework for data analysis and pattern recognition.

Traditional approaches to detecting plant diseases and pests have relied on image recognition techniques based on manually designed features. These approaches are often complex, requiring extensive expertise, and their performance frequently depends on subjective interpretation. DL provides an effective alternative by autonomously learning features from large-scale datasets without manual intervention. Multilayered models possess continuous learning and feature extraction capabilities, facilitating image classification and recognition in plant pathology. As the volume of training data and computational power increases, the representational capacity of DNNs correspondingly improves.

**Figure 1:**
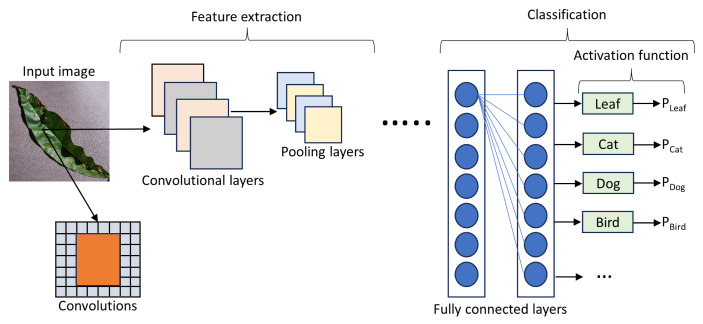
Structure of a convolutional neural network (CNN) for image classification.

In recent years, DL-based approaches, particularly CNN models, have gained prominence in plant disease and pest image recognition, providing more accurate and efficient solutions than traditional methods. As illustrated in Figure 1, a CNN model typically consists of several layers: an input layer, convolutional layers, pooling layers, fully connected layers, and an output layer. During a single iteration, convolutional and pooling layers perform a sequence of alternating operations. This process involves multiple alternating cycles between convolutional and pooling layers, producing a cascading effect. A distinctive property of CNN architecture is that neurons in convolutional and pooling layers are not fully connected.

The convolutional layer plays a key role in extracting salient features from input data through convolutional operations. These convolutional operations apply filters to detect spatial patterns and relationships within the input data. Subsequently, pooling layers reduce the spatial dimensions, decreasing computational cost while preserving critical feature information. The alternating structure of convolutional and pooling layers enhances the network’s capacity to capture complex hierarchical patterns in data (Barbedo, 2018). The connections between neurons in the convolutional and pooling layers are partial rather than fully connected, enhancing model efficiency. The connectivity paradigm increases network efficiency by selectively retaining essential information and discarding redundant details.

**Figure 2:**
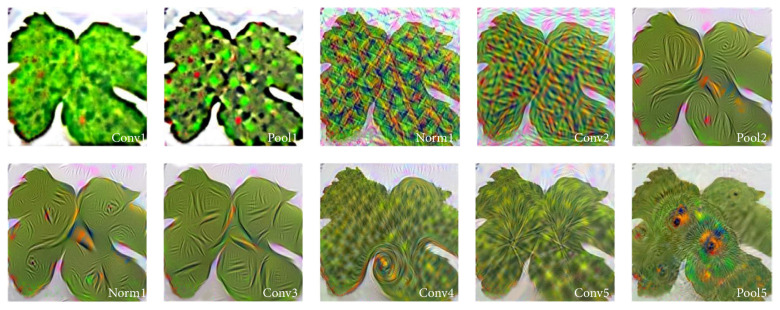
Visual representations of the output layers at each processing stage in the CaffeNet CNN, including convolution, pooling, and normalization layers, used for leaf-based plant disease identification. (adapted by Sladojevic et al., 2016).

Consider the visualization of leaf images in a CNN framework applied for plant disease identification. Figure 2 illustrates the progressive transformation of leaf images at each processing stage. This visual representation demonstrates how a CNN systematically extracts features and facilitates accurate classification in complex problem domains such as plant disease identification.

### 2.1. Architectures and tools

DL is characterized by the availability of well-established benchmark architectures that provide researchers with a solid foundation for model development, eliminating the need to build models from scratch. These architectures include AlexNet (Krizhevsky et al., 2012), CaffeNet (Jia et al., 2014), VGG (Simonyan and Zisserman, 2015), GoogleNet (Szegedy et al., 2015), and Inception-ResNet (Szegedy et al., 2017), among others, each offering distinct design principles and feature extraction capabilities. Furthermore, Canziani et al. (2017) evaluated and selected architectures based on their specific strengths and suitability for targeted applications and datasets. By pretraining on large-scale datasets such as ImageNet (Deng et al., 2009), neural network architectures acquire pretrained weights that facilitate feature learning and accelerate model convergence. Consequently, pretrained models can deliver accurate classifications across specific problem domains (Pan and Yang, 2010).

Researchers employing DL techniques utilize a variety of tools and platforms. The most widely used frameworks include TensorFlow, Keras, Theano, PyTorch, Caffe, Pylearn2, TFLearn, and the Deep Learning Toolbox in MATLAB. These frameworks provide a flexible environment for experimenting with various architectures and training models on diverse datasets. The individual functionalities of these open-source frameworks are summarized in Table 1. Theano and Caffe simplify implementation by integrating popular architectures such as AlexNet, VGG, and GoogleNet as built-in libraries or predefined classes. PyTorch and TensorFlow demonstrate high scalability through integration with third-party libraries and diverse network architectures, offering remarkable efficiency in training large-scale networks on graphics processing units (GPUs) and enabling accelerated training performance.

## 3. Applications of deep learning in plant disease detection

**Figure 3:**
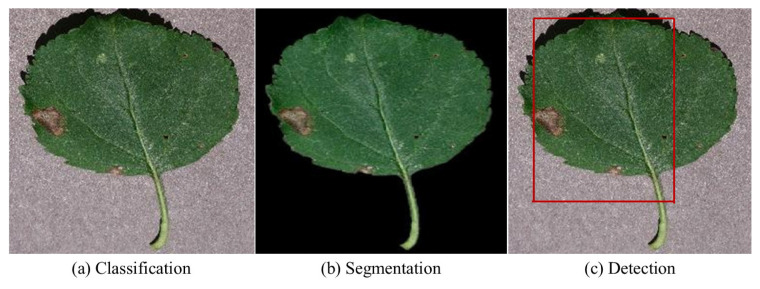
The three main deep learning approaches for plant recognition.

This section provides a comprehensive overview of methods employed to identify plant diseases and pests through DL applications. Figure 3 visually represents the major components of DL-based systems, including classification, detection, and segmentation. Additionally, the following subsections are organized according to the specific DL methods employed.

### 3.1. Feature extraction and selection

In the early stages of DL-based plant disease and pest classification research, researchers primarily relied on the inherent feature extraction capabilities of CNN models. These CNN-based feature extraction methods were combined with traditional classifiers to enhance overall classification performance (Singh et al., 2018). Input images are fed into a pretrained CNN to extract representative image features, which are subsequently passed to a conventional ML classifier for final classification.

Yalcin and Razavi (2016) proposed a CNN-based framework for image feature extraction, integrating support vector machine (SVM) classifiers with various kernels and feature descriptors such as local binary patterns (LBP) and global image descriptor (GIST). Similarly, Mohameth et al. (2020) developed CNN architectures incorporating transfer learning and deep feature extraction using the publicly available PlantVillage dataset. Their findings identified the SVM as the most effective classifier for leaf disease detection, demonstrating the potential of DL-based technological solutions in plant pathology.

Hasan et al. (2019) identified and classified nine rice diseases using features extracted from a deep CNN model, which were subsequently processed by a SVM classifier, achieving 97.5% accuracy. Transfer learning is a DL strategy that transfers knowledge acquired from previous tasks to improve learning efficiency on new tasks. In this approach, only specific layers of pretrained networks are fine-tuned with new datasets, substantially reducing the data requirements typically associated with ML tasks (Pan and Yang, 2010).

Sethy et al. (2020) developed deep CNNs for rice leaf disease identification through deep feature extraction. Using 5932 images representing four rice diseases—bacterial blotch, pat, brown spot, and tungro—the study evaluated eleven CNN models with transfer learning, integrating deep features with SVMs. The results indicated that SVM classifiers using deep features outperformed direct transfer learning approaches in terms of accuracy. The study also evaluated lightweight architectures such as MobileNetV2 and ShuffleNet. Evaluation metrics included accuracy, sensitivity, specificity, false positive rate (FPR), F1-score, and training time. The highest performance was achieved using ResNet50-derived features with an SVM classifier, yielding an F1-score of 0.9838.

Ramesh et al. (2018) employed the random forest (RF) algorithm for papaya leaf classification and compared its performance with other classifiers, including logistic regression (LR), classification and regression tree (CART), SVM, k-nearest neighbors (KNN), and naive Bayes. For object recognition, they employed histogram of oriented gradients (HOG) for feature extraction, incorporating color histograms, Haralick texture features, and Hu moments. The resulting feature vector was subsequently used for classification. The results indicated that the RF algorithm achieved the highest accuracy of 70.14%.

Islam et al. (2017) employed a multiclass SVM classifier to analyze potato leaf images, distinguishing between early blight, late blight, and healthy samples. The dataset consisted of 200 diseased and 100 healthy leaf images. The gray-level co-occurrence matrix (GLCM) method was used to extract 10 texture and color features. A linear-kernel SVM was trained on 180 images and tested on 120, with performance evaluated using precision, accuracy, F1-score, and recall metrics. The model achieved 95% accuracy with a 60–40 train–test split, and 93.7% accuracy using five-fold cross-validation. The results demonstrate the effectiveness of multiclass SVM classifiers for potato disease classification.

Recent advances in DL have introduced architectures beyond CNNs, including transformer-based models such as the vision transformer (ViT) and swin transformer (Khan et al., 2023a). These architectures employ self-attention mechanisms instead of convolutional operations, enabling them to better capture the global dependencies inherent in plant disease images. Studies have shown that ViT models perform better when trained on large-scale datasets with high-resolution images, whereas CNNs remain more efficient for smaller datasets (Ogrekci et al., 2023).

Additionally, generative adversarial networks (GANs) have been employed to synthesize artificial images, thereby enhancing the generalization capability of plant disease classification models (Bi and Hu, 2020). Multimodal DL approaches combining hyperspectral and thermal imaging have also been employed to detect early-stage infections before visible symptoms appear (Terentev et al., 2022).

These developments underscore the continuous advancement of DL techniques in plant disease detection. They emphasize the importance of integrating diverse feature extraction techniques, leveraging pretrained models, and adopting alternative network architectures to achieve higher accuracy and robustness in practical agricultural applications.

### 3.2. Disease classification

Image classification represents a fundamental task within the field of image processing. It has played a crucial role in the advancement of computer vision, with major breakthroughs achieved through the adoption of DL models and novel approaches. Numerous studies have employed various methods to identify and classify plant diseases. This section presents a detailed analysis of plant disease image classification approaches, further elaborated in the following subsections.

#### 3.2.1. Classification with visualization techniques

In the following studies, visualization techniques were integrated with DL methods to enhance interpretability and achieve a deeper understanding of disease characteristics. Brahimi et al. (2018) employed a saliency map-based visualization technique to classify disease symptoms in affected plants. The authors utilized the CaffeNet CNN architecture to identify 13 plant diseases, outperforming existing models for plant disease classification. The proposed model achieved an accuracy of 99.76%. Atila et al. (2021) introduced the EfficientNet architecture and compared its performance with other state-of-the-art models using the PlantVillage dataset. They applied visualization techniques to augment and interpret the dataset more effectively. Training was performed on both the original and augmented datasets using transfer learning, with all layers set as trainable. The model achieved accuracy rates of 99.91% and 99.97% on the original and augmented datasets, respectively. Nagasubramanian et al. (2019) employed a three-dimensional deep convolutional neural network (DCNN) model along with saliency map visualization to distinguish between healthy and infected soybean leaves affected by blight. The classification model achieved an accuracy of 95.73%.

Feng et al. (2021) utilized hyperspectral imaging to rapidly and accurately detect rice diseases across four distinct rice varieties. Because developing a separate classifier for each variety is time- and resource-intensive, the study adopted deep transfer learning to enhance detection performance across rice varieties. A total of three deep transfer learning approaches were evaluated. Among these, fine-tuning exhibited the highest transferability, achieving accuracy rates above 88% on test sets for most transfer tasks. The deep CORAL method also performed well, achieving over 80% accuracy across four tasks and outperforming the deep domain confusion (DDC) approach. Additionally, a multitask transfer learning strategy produced promising results, demonstrating the effectiveness of both pairwise and multitask transfer mechanisms. Saliency maps were employed to visualize the primary wavelength ranges identified by the CNN, both with and without transfer learning, revealing substantial overlap in the captured spectral features.

Gonzalez-Huitron et al. (2021) investigated the training and evaluation of four CNN models developed to identify diseases in tomato leaves. The study employed the PlantVillage dataset, consisting of 18,160 red–green–blue (RGB) images categorized into 10 distinct classes for transfer learning. The selected CNN models incorporated depthwise separable convolutional architectures optimized for deployment on low-power devices. Model performance was assessed both quantitatively and qualitatively using standard evaluation metrics and saliency maps to enhance interpretability. To demonstrate the practical applicability of their method, the authors developed a graphical user interface (GUI) enabling user interaction with the system.

Cao et al. (2022) investigated early detection of rice bacterial leaf blight (BLB) infections using hyperspectral imaging combined with a spectral dilated convolutional 3D CNN (SDC-3DCNN). Hyperspectral images captured from infected rice leaves during the tillering stage were preprocessed using Savitzky–Golay smoothing. Wavelengths between 450 and 950 nm were selected, and features extracted using principal component analysis (PCA) and RF algorithms were input into the SDC-3DCNN model. The model’s performance was evaluated using different input features and spectral dilation ratios.

Furthermore, saliency maps were used to visualize the sensitivity of individual wavelengths, providing insight into the model’s focus regions and interpretability. Experimental results indicated that the SDC-3DCNN model achieved 95.44% accuracy when using 50 characteristic wavelengths identified by the RF algorithm with a dilation ratio of 5. Saliency analysis identified sensitive wavelength bands within the 530–570 nm range, corresponding to the critical wavelengths extracted by the RF algorithm. These findings underscore the effectiveness of integrating hyperspectral imaging with DL for early detection of asymptomatic rice BLB infections, offering critical support for early warning systems and preventive measures in rice cultivation.

Anim-Ayeko et al. (2023) proposed a ResNet-9 model for classifying late blight in potato and tomato leaf images. The PlantVillage dataset, initially containing 3990 training samples, was augmented to improve model generalization capability. After data augmentation, an extensive hyperparameter optimization process was performed to determine the optimal configuration for model training. The optimized ResNet-9 model was subsequently trained and evaluated on a test set comprising 1331 images. Experimental results demonstrated high classification performance, achieving 99.25% test accuracy, 99.67% precision, 99.33% recall, and an F1-score of 99.33%.

Sun et al. (2019) proposed an image analysis approach for detecting leaf diseases in tea plants, involving superpixel block generation, key-point detection, and extraction of blurred contours of salient regions. Texture features were removed, and a classification map was generated using a SVM classifier. Morphological and algebraic operations were applied for block restoration, resulting in a highly accurate saliency map. The method was evaluated on 261 diseased leaf images, achieving 98.5% accuracy, 96.8% precision, 98.6% recall, and a 97.7% F1-score.

In a related study, Tembhurne et al. (2023) developed a classification model based on the MobileNet architecture, augmented with additional hidden layers and fine-tuned using saliency map-based optimization. The model was trained on a dataset containing 12,318 images and designed to classify inputs into 64 distinct categories across 22 plant species. Experimental results showed that the proposed model achieved 95.94% classification accuracy, underscoring its effectiveness for multiclass plant disease detection.

Khan et al. (2020) outlined a five-step process for classifying cucumber leaf diseases, consisting of infected spot segmentation, image enhancement, feature selection, deep feature extraction, and classification. Image enhancement was applied to boost local contrast, making infected regions more distinguishable. For segmentation, a novel approach combining Sharif’s saliency method with active contour segmentation was employed. Pretrained models, specifically VGG-19 and VGG-M, were used for feature extraction, with the most relevant features selected based on local entropy and standard deviation. The resulting features were subsequently fed into a multiclass support vector machine (SVM) classifier to identify the disease, achieving a classification accuracy of 98.08%.

Additionally, some researchers have employed heatmap visualization, a common DL interpretability tool that helps analyze neural network outputs, particularly in prediction and segmentation tasks. These visualizations assist analysts and researchers by identifying regions of interest that the model focuses on. Wiesner-Hanks et al. (2019) applied a heatmap-based visualization approach to accurately outline maize disease lesion contours. Using images captured by unmanned aerial vehicles (UAVs), their model achieved exceptional accuracy in classifying lesions, even at the millimeter scale. The achieved accuracy rate of 99.79% represents the highest reported performance for aerial plant disease classification to date. Albattah et al. (2022) proposed an improved CenterNet framework integrated with a DenseNet-77 backbone. The proposed framework consisted of three main steps: creating region-of-interest (ROI) annotations, applying the enhanced CenterNet with DenseNet-77 for feature extraction, and using CenterNet for plant disease detection and classification.

DeChant et al. (2017) introduced an innovative computational pipeline utilizing convolutional neural network (CNN) models to address challenges associated with limited data and irregularities in field-grown plant images. The approach involved training multiple CNNs to classify small image patches displaying northern leaf blight (NLB) lesions. Predictions from these networks were aggregated into heatmaps, which were then used as input to train a final CNN that classified entire images as diseased or healthy. The system achieved a classification accuracy of 96.7% on a test dataset comprising images not used during training.

Nawaz et al. (2024) proposed a CenterNet-based methodology integrating a ResNet50 backbone with a spatial channel attention mechanism to extract deep, disease-specific features from image samples. This enhanced feature extraction process was implemented within the single-stage detection architecture of the CenterNet framework. The developed model, named CoffeeNet, was evaluated using the Arabica coffee leaf dataset, which contains images captured under realistic and complex environmental conditions. Experimental results showed that CoffeeNet achieved 98.54% classification accuracy and a mean average precision (mAP) of 0.97, demonstrating strong robustness in real-world applications.

In a related study, Kumar et al. (2022) developed an automated detection system for three major corn diseases: common rust, Cercospora spot, and northern leaf blight (NLB). The system combined image recognition techniques with DL methods, specifically employing Faster R-CNN with a ResNet50 backbone network. The model achieved 93.5% classification accuracy when evaluated on real-time imaging data. Additionally, Chowdhury et al. (2021) employed the EfficientNet architecture to classify 18,161 tomato leaf images using a modified U-Net framework. This model achieved 98.66% accuracy, with the EfficientNetB7 variant outperforming others—reaching 99.95% in binary classification and 99.12% in six-class classification tasks using segmented images.

In cases where certain diseases manifest in early stages without visible symptoms, indicative signs may appear in regions of the electromagnetic spectrum invisible to the human eye. In such scenarios, hyperspectral and multispectral imaging techniques prove highly beneficial. For instance, advanced imaging techniques enable the detection and analysis of subtle variations in electromagnetic wavelengths, offering insights that would otherwise remain undetected through conventional visual observation.

Park et al. (2018) proposed a hyperspectral image analysis method utilizing the minimum redundancy maximum relevance (mRMR) algorithm to select the most informative spectral bands. By integrating a CNN with a fully connected network (FCN), the proposed deep neural architecture effectively classified dimensionally reduced hyperspectral data. The approach successfully identified five key spectral bands, achieving higher classification accuracy than conventional RGB imaging. In a related study, Polder et al. (2019) employed hyperspectral imaging to detect Potato virus Y (PVY) infections in seed potatoes. Hyperspectral images were acquired during field experiments using a line-scan hyperspectral camera with a spatial resolution of 5 mm. The study utilized two CNNs for model training, using data collected from two independent field experiment series.

Nguyen et al. (2021) applied hyperspectral imaging at the plant level to detect and classify grapevines infected with the grapevine vein clearing virus. The experiments involved both pixel-by-pixel and image-level classification approaches. The methodology combined DL and ML architectures for analysis. The RF classifier exhibited superior performance in both pixel-level and image-level classification, particularly in scenarios involving high-dimensional feature spaces. Xu et al. (2023) proposed residual feedback ensemble-CNN (RFE-CNN), an integrated DL framework combining residual channel attention blocks (RCAB), feedback blocks (FB), enhanced memory layers (EML), and CNNs. A pair of parallel CNNs was used for feature extraction, optimized via RCAB, iteratively trained using FB, and subsequently processed with CNN and EML for final classification. RFE-CNN outperformed benchmark models, demonstrating superior time efficiency, recognition accuracy, and adaptability, achieving an overall accuracy of 98.83%.

Yong et al. (2023) focused on automated detection of basal stem rot (BSR) at the seedling stage using pretrained DL models and hyperspectral imaging. Aerial images of oil palm seedlings were segmented into three distinct regions to assess spectral differences across various leaf positions. To evaluate the impact of background imagery on recognition accuracy, seedling images were automatically segmented using a region-based CNN (R-CNN) incorporating mask region proposals. Gao et al. (2023) proposed a multidimensional CNN framework integrating spectral, spatial, and spectral–spatial features for potato disease detection. The framework integrates a 1D-CNN for spectral feature extraction, a 2D-CNN for spatial representation learning, and a 3D-CNN for spectral–spatial data analysis. Convolution operations in the 1D-CNN and 2D-CNN were optimized to minimize data loss during processing. Experimental evaluations on real potato disease datasets achieved a detection accuracy of up to 99.87%, confirming the model’s effectiveness.

These visualization techniques form integral components of the broader discipline of explainable artificial intelligence (XAI)—a rapidly evolving field aimed at improving the interpretability, transparency, and reliability of complex ML models, particularly those employing DL architectures. In agriculture, the implementation of XAI methodologies plays a pivotal role in enhancing the reliability of automated decision-making processes, including disease diagnosis and crop management. By allowing agronomists and stakeholders to better understand how specific features or patterns influence model predictions, XAI facilitates improved validation and broader acceptance of these technologies. Moreover, visualization-based classification methods constitute a key subset of XAI strategies specifically designed for plant disease detection, where visual interpretability is essential. These techniques not only assist in localizing and identifying diseased regions within plant imagery but also promote transparent interaction between algorithms and end-users, thereby increasing confidence in automated agricultural diagnostics.

**Figure 4:**
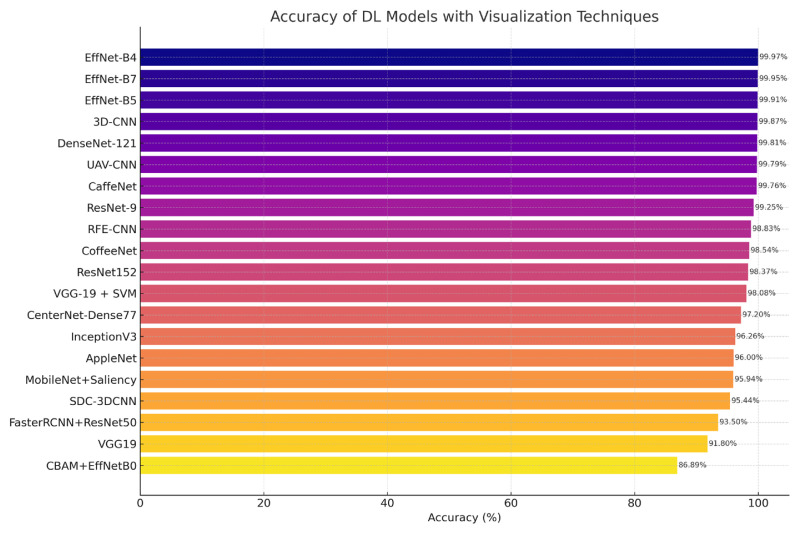
Accuracy of deep learning models using visualization techniques for plant disease classification.

Figure 4 provides a comparative summary of classification accuracies achieved various DL models utilizing visualization techniques. These methods include saliency maps, heatmaps, hyperspectral imaging, and spectral attention mechanisms, each applied to distinct datasets. The figure highlights the superior performance of EfficientNet-, DenseNet-, and CenterNet-based architectures in achieving peak accuracy, with several models exceeding the 99% threshold.

#### 3.2.2. Classification without visualization techniques

Applying DL techniques for plant disease classification without visualization methods represents a novel direction in agricultural diagnostics. Traditional diagnostic methods often rely on visual inspection of symptoms, which are constrained by the observer’s expertise and environmental conditions. In contrast, DL models can be trained on diverse datasets that include genetic and phenotypic information related to plant diseases. This enables models to learn complex patterns and associations, allowing disease identification based on nonvisual features. This approach provides a more comprehensive and precise means of diagnosing diseases, reducing dependence on visual cues and proving particularly valuable when direct observation is limited.

Naik et al. (2022) conducted a detailed study to classify five chili diseases using 12 CNN models. The analysis included 12 models, namely EfficientNetB0, InceptionV3, AlexNet, DarkNet53, ShuffleNet, SqueezeNet, DenseNet201, ResNet101, VGG19, MobileNetV2, NasNetLarge, and XceptionNet. The VGG19 model demonstrated the highest performance, achieving 83.54% accuracy without data augmentation or visualization techniques.

Dai et al. (2024) proposed a multilevel deep feature fusion network, termed deep feature network with pyramidal squeezed attention network (DFN-PSAN) for plant disease classification. The architecture integrates the YOLOv5 backbone and neck components, leveraging their strengths in feature extraction. The model further incorporates pyramidal squeezed attention (PSA) mechanisms and multiple convolutional layers to enhance the overall performance of the PSAN framework. This network effectively fuses and processes multilevel deep features from the DFN, while the PSA module provides pixel-level attention to highlight disease-related regions in plant images. Experiments on three plant disease datasets showed that the DFN-PSAN framework achieved an average accuracy and F1-score of 95.27%, underscoring its effectiveness in accurate plant disease diagnosis.

Dey et al. (2022) compared the performance of pretrained deep CNN models for classifying rice diseases such as Hispa, brown spot, leaf blight, and Nitrogen, Phosphorus, and Potassium (NPK) deficiency symptoms. Training the DL models on different combinations of public datasets revealed that mixed datasets yielded the best results, with the VGG19 model achieving 91.8% accuracy. The study demonstrated that a simple, well-structured CNN outperformed more complex models in classifying phosphate-deficient leaves.

Vallabhajosyula et al. (2022) introduced an automated plant disease classification framework, termed deep ensemble neural networks (DENN), which leverages transfer learning using pretrained models. To reduce overfitting and enhance model robustness, various data augmentation techniques—including image enhancement, rotation, scaling, and translation—were employed. The proposed framework was evaluated using the PlantVillage dataset, which includes 38 classes spanning 14 crop species. Faisal et al. (2023) aimed to improve classification accuracy for citrus plant datasets by employing state-of-the-art transfer learning architectures. Their methodology incorporated convolutional neural networks (CNNs) with pretrained architectures, including EfficientNetB3, ResNet50, MobileNetV2, and InceptionV3, focusing primarily on disease detection and categorization in citrus plants.

Alirezazadeh et al. (2023) examined the impact of integrating the convolutional block attention module (CBAM) on improving plant disease classification performance in CNN architectures. CBAM—a lightweight attention mechanism—was embedded into well-established CNN architectures. The models were fine-tuned on the DiaMOS Plant dataset, and experimental results showed that EfficientNetB0 combined with CBAM achieved the highest classification accuracy (86.89%), outperforming the standalone EfficientNetB0 model. Andrew et al. (2022) proposed the use of pretrained CNN models for plant disease classification, utilizing the PlantVillage dataset. Among the evaluated models, DenseNet121 achieved superior performance, with a classification accuracy of 99.81%.

Amin et al. (2022) proposed a model employing pretrained CNNs, specifically DenseNet121 and EfficientNetB0. These models were used to extract features from corn leaf images. Data augmentation techniques expanded the training dataset by introducing image variations, enabling the model to capture complex patterns. The proposed model achieved a classification accuracy of 98.56%, outperforming ResNet152 and InceptionV3, which require higher computational resources. Specifically, the proposed model outperformed ResNet152 and InceptionV3, which achieved accuracies of 98.37% and 96.26%, respectively.

Coulibaly et al. (2019) introduced a transfer learning-based approach for identifying mildew diseases in pearl millet. They employed the classical VGG16 CNN model, pretrained on the publicly available ImageNet dataset. The experimental results demonstrated strong performance, with an accuracy of 95% and a recall of 94.5%. In another study employing VGG architectures, Oppenheim et al. (2019) used potato images captured under natural lighting conditions, encompassing diverse sizes, shades, and shapes. The classification of infected potato images was achieved through fine-tuning of the VGG network. Experimental results showed that both transfer learning and training from scratch produced successful classification outcomes.

Fan et al. (2022) proposed a methodology integrating transfer learning-based deep feature extraction, traditional handcrafted features, and center loss to enhance discriminative capability. Experimental evaluations on three datasets—two apple leaf and one coffee leaf—achieved classification accuracies of 99.79%, 92.59%, and 97.12%, respectively. Fraiwan et al. (2022) employed the InceptionResNetV2 convolutional neural network (CNN) combined with transfer learning to identify rice leaf diseases. After parameter optimization tailored to the classification task, the proposed model achieved an accuracy of 95.67%.

Rancon et al. (2019) employed transfer learning-based feature extraction techniques to classify vine plant diseases. The research was conducted across two vineyards in the Bordeaux region of France (Aquitaine), where images of healthy and diseased vine plants were collected during the summer of 2017. The collected images were meticulously annotated at the leaf level, resulting in a dataset of approximately 6000 images (224 × 224 pixels) categorized into red and white cultivars. For classification, the authors compared the efficacy of scale-invariant feature transform (SIFT) encoding with that of pretrained DL feature extractors. The highest overall accuracy of 91% was achieved using MobileNet feature representations.

Ahmad et al. (2021) introduced a sequential transfer learning methodology designed to accelerate convergence, mitigate overfitting, and prevent negative transfer across diverse domains. The proposed system was evaluated on two distinct plant disease datasets: the PlantVillage dataset and a more challenging pepper disease dataset. Results showed classification accuracies of 99% on the pepper dataset and 99.69% on the PlantVillage dataset, demonstrating the approach’s robustness across domain contexts. Abbas et al. (2021) developed a DL framework for tomato disease detection, leveraging a conditional generative adversarial network (C-GAN) to generate synthetic tomato leaf images. Subsequently, the DenseNet121 model was fine-tuned through transfer learning on both synthetic and real image datasets to classify tomato leaf images into 10 disease categories.

Hassan et al. (2021) demonstrated improved computational efficiency by replacing standard convolutional layers with depthwise separable convolutions. The models were trained on an open dataset comprising 14 plant species and 38 disease categories, including healthy samples. Several hyperparameters—batch size, dropout rate, and number of epochs—were systematically optimized to evaluate model performance, yielding promising results.

Chen et al. (2021) employed MobileNetV2 pretrained on ImageNet and incorporated an attention mechanism to capture interchannel dependencies and spatial feature salience. They further optimized the loss function and applied a two-stage transfer learning approach during training. This proposed method outperformed existing approaches, achieving an average identification accuracy of 99.67% on a publicly available plant disease dataset. Simhadri and Kondaveeti (2023) employed a transfer learning strategy using 15 pretrained CNN models for automatic identification of rice leaf diseases. Among the models evaluated, InceptionV3 achieved the highest performance. Nagaraju and Chawla (2023) proposed a novel deep CNN architecture, NPNet-19, explicitly designed for maize plant disease classification. The model was evaluated on an expanded dataset comprising 15,960 images across six disease categories and one healthy class, primarily collected from maize fields in Telangana. During training, NPNet-19 achieved 97.51% accuracy, while testing yielded 88.72% accuracy, reflecting consistent yet realistic generalization performance.

Dong et al. (2023) proposed a set of widely used pretrained models aimed at improving the diagnostic accuracy of plant disease image classification. The models were evaluated across multiple plant disease diagnosis tasks. Experimental results showed that the pretrained models outperformed existing architectures in terms of accuracy while requiring less training time. Assad et al. (2023) introduced AppleNet, a DL-based multiclass classification model for apple plant diseases. The model efficiently extracted features from a real-world dataset using transfer learning with the pretrained ResNet50 CNN on the ImageNet dataset, thereby saving computational resources and time. By fine-tuning hyperparameters and training on 2897 augmented images, AppleNet achieved a classification accuracy of 96.00% for apple disease identification. Pradhan et al. (2022) conducted a comparative study of 10 widely recognized CNN architectures to evaluate their effectiveness in apple disease classification. Results demonstrated that DL techniques achieved high classification accuracy, with DenseNet201 outperforming all other models by attaining 98.75% accuracy.

Ahad et al. (2023) examined the rice disease classification capabilities of six CNN-based architectures. Transfer learning was explicitly applied, and an ensemble model named DEX was developed, integrating DenseNet121, EfficientNetB7, and Xception architectures. The evaluation employed a dataset comprising nine common rice diseases prevalent in Bangladesh. Sood and Singh (2023) applied transfer learning to pretrained CNN models—VGG16, ResNet50, InceptionV3, and DenseNet121—for plant disease classification using the PlantVillage dataset. Their findings underscored the effectiveness of transfer learning in leveraging existing pretrained models to enhance classification performance in agricultural applications.

Barbedo (2018) examined how dataset size and diversity influence the effectiveness of DL techniques in plant pathology. The investigation used an image database containing 12 plant species, each with varying sample counts, disease frequencies, and environmental conditions. Nader et al. (2022) compared the performance of several pretrained CNN models using transfer learning and ensemble learning strategies. Their methodology employed ensemble learning, combining three widely recognized CNN architectures to enhance classification accuracy for grape disease detection.

Adnan et al. (2023) proposed a method employing pretrained deep CNNs to classify a diverse dataset comprising 52 categories of plant diseases and healthy leaf samples. The study evaluated models including Xception, ResNet50, InceptionResNetV2, and InceptionV3, integrated with EfficientNetB3 and an adaptive augmented DL strategy, achieving high accuracy across multiple disease categories. Hayit et al. (2023) investigated the use of pretrained CNN models for early detection of Fusarium wilt infection types in chickpeas. Using a novel dataset of infected chickpea plants, the DenseNet201 model achieved an average test accuracy of 90%.

Zia Ur Rehman et al. (2021) employed two pretrained DL models combined with image enhancement and augmentation techniques to expand the citrus disease dataset. Hybrid contrast stretching was applied to enhance visual quality, followed by transfer learning to fine-tune the models. Additionally, feature fusion and optimization using the whale optimization algorithm (WOA) were applied to select the most relevant features, achieving a classification accuracy of 95.7% across six citrus disease categories. Chen et al. (2020) likewise applied transfer learning in their study. They employed pretrained models—VGGNet and Inception—originally trained on the ImageNet dataset. Instead of random weight initialization, the researchers leveraged knowledge from these pretrained networks. Their model demonstrated significantly improved performance compared to other established techniques.

Arshaghi et al. (2023) employed CNN-based approaches to classify potato diseases. Using a dataset of 5000 potato images, the proposed DL method was evaluated against established architectures, including GoogLeNet, AlexNet, R-CNN, VGG, and transfer learning frameworks. Results indicated that the proposed method outperformed existing models, achieving classification accuracies of up to 100%.

Thangaraj et al. (2021) aimed to enhance tomato plant disease detection by investigating the impact of different optimizers within a transfer learning framework. The system was evaluated using both real-time and stored tomato leaf images. Performance evaluations using Adam, SGD, and RMSprop optimizers revealed that the transfer learning approach was the most effective for automated classification of tomato leaf diseases. Uguz and Uysal (2021) explored the application of transfer learning using VGG16, VGG19, and a custom CNN architecture. A primary objective of their study was to examine the impact of data augmentation on model performance. Results indicated that models trained with data augmentation achieved up to 95% accuracy, compared to 88% for models trained without augmentation.

Some studies have integrated ML or optimization algorithms with DL models. Abd Algani et al. (2023) developed a DL technique, ant colony optimization-convolutional neural network (ACO-CNN), for plant leaf disease classification. Using ant colony optimization (ACO), the system extracted color, texture, and leaf features from images via a CNN-based classifier. The proposed approach outperformed existing methods, demonstrating higher diagnostic accuracy across multiple evaluation metrics. Alsubai et al. (2023) presented a hybrid DL model incorporating improved salp swarm optimization (ISSO). The method classified grape leaf images into four categories, beginning with median filtering (MF) for noise removal during preprocessing. The framework employed a dilated residual network (DRN) for feature extraction and utilized the Adam optimizer. Additionally, a convolutional neural network-gated recurrent unit (CNN-GRU) hybrid model was used for disease classification.

Verma et al. (2023) proposed a metalearning framework for plant disease classification, recommending top-n models for unseen datasets based on benchmark evaluations. Framework performance was evaluated using rank-biased overlap (RBO) to compare predicted and actual rankings. Extensive experiments with different metaextractor and metalearner configurations revealed that a probe network trained for 10 epochs, using standard deviation as the metaextractor and support vector regression (SVR) as the metalearner, outperformed other configurations. Garg and Alam (2022) proposed a hybrid architecture combining a pretrained CNN with a long short-term memory (LSTM) network. Transfer learning was applied to extract deep features from the fully connected layers of pretrained models, including InceptionV3, VGG16, and Xception. These features were concatenated with LSTM outputs and passed through a fully connected layer to enhance the model’s attention to salient information. The integrated model was then applied to classify apple foliar diseases.

Chug et al. (2023) introduced an innovative hybrid framework integrating ML and DL, featuring 40 hybrid DL models. These models incorporate eight pretrained EfficientNet variants (B0–B7) as feature extractors, coupled with ML classifiers including RF, LR, KNN, AdaBoost, and stochastic gradient boosting. Tabbakh and Barpanda (2023) designed a hybrid model named TLMViT, which combines transfer learning and vision transformer (ViT) architectures for plant disease identification. The TLMViT framework includes data collection using the PlantVillage and wheat datasets, image augmentation to reduce overfitting, feature extraction based on pretrained and ViT models, and final classification through a multilayer perceptron (MLP) classifier. Altalak et al. (2022) proposed a tomato leaf disease classification model that combines a CNN with a CBAM and a SVM.

Bajait and Malarvizhi (2023) proposed a grape leaf disease classification framework consisting of several key stages. The process begins with image preprocessing, including contrast enhancement using contrast limited adaptive histogram equalization (CLAHE) and noise reduction with adaptive bilateral filtering (ABF). Feature extraction was performed using the SqueezeNet model, and hyperparameter optimization was conducted with the equilibrium optimizer (EO) algorithm. Classification was subsequently carried out using a stacked autoencoder (SAE) model. Simulation analysis of the EODTL-GLDC technique on newly introduced plant disease datasets demonstrated highly promising results.

**Figure 5:**
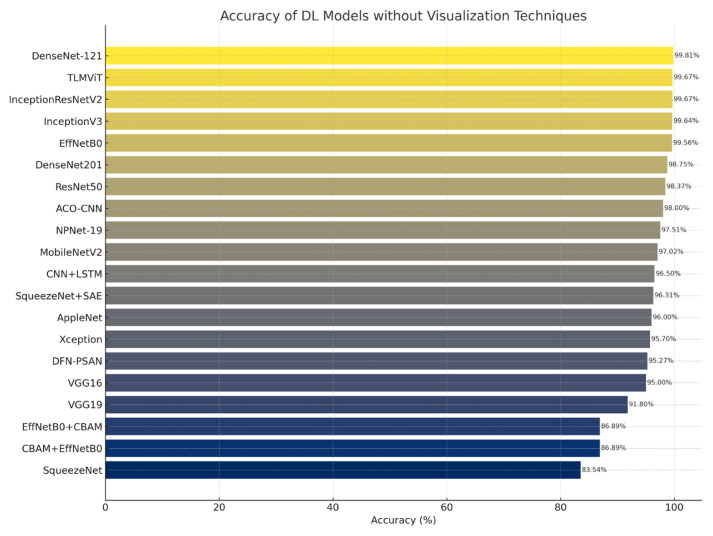
Accuracy of deep learning models without visualization techniques for plant disease classification.

Figure 5 illustrates the performance of DL models applied to plant disease classification in the absence of visualization-based techniques. These models rely exclusively on raw image data and transfer learning architectures, excluding interpretable components such as saliency maps or class activation overlays. Notably, several models, including DenseNet121, EfficientNetB0, and InceptionResNetV2, achieved over 99% classification accuracy, underscoring the robustness of pure CNN-based pipelines. This finding highlights that high-performance plant disease diagnosis is achievable even without visualization-enhanced interpretability methods.

Tables 2 and 3 summarize the reviewed studies, highlighting the key models, accuracy rates, and principal contributions.

Studies employing visualization techniques—such as saliency maps, heatmaps, and feature attention mechanisms—have shown notable improvements in both interpretability and accuracy. Brahimi et al. (2018) employed saliency maps with the CaffeNet architecture, achieving 99.76% accuracy, whereas Atila et al. (2021) leveraged EfficientNetB5/B4 with augmented datasets, achieving up to 99.97% accuracy.

Other studies, including Nagasubramanian et al. (2019) and Wiesner-Hanks et al. (2019), explored 3D-DCNN architectures and UAV-based CNN classification, demonstrating that incorporating heatmaps and hyperspectral imaging enhances disease localization. These visualization-based methods provide an additional layer of interpretability, making them valuable for automated diagnostics and human-in-the-loop decision-making.

Studies focusing on direct classification without visualization techniques have emphasized optimizing model architectures, feature selection strategies, and training procedures to enhance performance. Naik et al. (2022) compared 12 CNN models, with VGG19 achieving 83.54% accuracy in chili disease classification. Several studies applied transfer learning, fine-tuning pretrained models such as ResNet, EfficientNet, MobileNet, and DenseNet for plant disease classification. Ahad et al. (2023) proposed an ensemble approach combining DenseNet121, MobileNetV2, and ResNet152V for rice disease detection, whereas Hassan et al. (2021) integrated depthwise separable convolutions, achieving up to 99.56% accuracy. Dai et al. (2024) introduced multilevel feature extraction methods, such as DFN-PSAN, to emphasize key disease regions in plant images, thereby enhancing classification robustness.

Several hybrid approaches integrate CNNs with metalearning and optimization algorithms to improve model adaptability. Garg and Alam (2022) developed a CNN–LSTM hybrid model for apple disease classification, leveraging sequential feature dependencies. Likewise, Abd Algani et al. (2023) applied ant colony optimization (ACO) in conjunction with CNNs to enhance color and texture feature extraction for leaf disease detection. Other metalearning strategies, such as the TLMViT model proposed by Tabbakh and Barpanda (2023), fused vision transformers (ViTs) with CNNs, enhancing the connection between spatial and contextual disease representations.

Despite substantial progress in DL-based plant disease classification, several challenges persist. Studies focusing on direct classification frequently encounter dataset biases and domain shifts, wherein models trained on controlled datasets often generalize poorly under real-world field conditions. In contrast, visualization-based methods—while offering improved interpretability—introduce computational overhead, rendering them less suitable for real-time deployment in resource-constrained environments. Future research should explore hybrid architectures that integrate self-supervised learning, generative adversarial networks (GANs), and multimodal fusion approaches—such as combining RGB and hyperspectral modalities—to overcome these limitations. Moreover, the integration of explainable artificial intelligence (XAI) frameworks can enhance model transparency and foster greater trust in DL-driven agricultural diagnostics.

### 3.3. Disease detection

Object detection in plant disease identification extends beyond simple classification to the complex task of spatial localization. In this domain, DL-based detection methods have evolved into two primary architectural paradigms: two-stage and one-stage networks.

One-stage detectors—such as the single shot multibox detector (SSD) and you only look once (YOLO)—have gained prominence due to their balance between inference speed and detection accuracy. Recent studies (Yin et al., 2024; Ali et al., 2024) have demonstrated the effectiveness of YOLO variants in detecting plant diseases, achieving high precision rates. Furthermore, integrating transformer-based architectures into detection frameworks has yielded promising results across diverse agricultural applications.

#### 3.3.1. One-stage detection networks

The removal of the traditional region proposal stage has led to a paradigm shift in the development of single-stage object detection algorithms. These methods streamline the classification and regression processes by integrating the detection head directly within the backbone network. This integration significantly enhances the inference speed of the detection network. Among single-stage detection frameworks, two prominent models—SSD and YOLO—are widely recognized. Both models share a common characteristic, utilizing the entire image as input to the network. Consequently, this approach demonstrates the capability of one-stage detection algorithms to perform complex tasks—such as object localization and classification—both rapidly and accurately.

Compared with conventional CNN architectures, the SSD employs VGG16 as its backbone and integrates a feature pyramid network (FPN) to extract multiscale features, thereby improving prediction accuracy. Qiang et al. (2023) developed a dual-backbone enhanced single shot multibox detector (SSD) model for identifying citrus diseases. The detection accuracy and recall of the model were assessed, and its robustness was verified through detailed analysis of the results. The numerical results indicated that the trained network achieved an mAP of 72.54% on the test dataset. Furthermore, the model attained an mAP of 86.01%. Guo et al. (2021) proposed a precise identification method for common tomato diseases using DL. Their approach features a multi-resolution detector enhanced with optimized bounding box generation and assignment to improve feature extraction. The inclusion of dropout and the ADAMW optimizer effectively reduces overfitting. The detector was trained on images of healthy and diseased tomatoes and reliably detects 10 different diseases.

Saleem et al. (2022) developed a transfer learning-based approach to enhance plant disease detection through optimized network weights. The study compared multiple DL architectures to evaluate performance improvements. Weight optimization yielded a 9.25% improvement in mean average precision (mAP), achieving an overall mAP of 91.33%. In an earlier study, Saleem et al. (2020) evaluated three DL metaarchitectures—SSD, Faster R-CNN, and R-FCN—within the TensorFlow object detection framework for plant leaf disease recognition. Among these, the SSD model trained with the Adam optimizer achieved the highest mAP of 73.07%, demonstrating superior disease detection capability.

Sun et al. (2020) proposed an enhanced SSD framework for detecting corn leaf spot disease under complex environmental conditions. Their method emphasizes multiscale feature fusion through CNNs. The pipeline comprises several stages, including data preprocessing, feature fusion, feature sharing, and final disease detection. The enhanced model substantially outperformed the original SSD, increasing mean average precision (mAP) from 71.80% to 91.83%.

Similarly, Bao et al. (2023) developed the adaptive spatial feature fusion network (ASFFNet) for detecting wheat scab. ASFFNet incorporates a feature enhancement module that combines global and local representations, thereby strengthening the network’s representational capacity. An adaptive feature fusion module subsequently merges these enhanced features across multiple scales, effectively addressing detection challenges arising from small disease regions and improving detection accuracy. Comparative evaluations demonstrated that ASFFNet outperformed state-of-the-art object detection algorithms—including SSD, RetinaNet, YOLOv3, and YOLOv4—in terms of average precision (AP).

In contrast, YOLO reconceptualizes object detection as a single-stage regression task, enabling end-to-end prediction through a unified CNN architecture. Leveraging global image context, YOLO directly predicts bounding boxes and class labels, substantially increasing detection speed while maintaining high accuracy. A summary of these studies is presented in Table 4.

#### 3.3.2. Two-stage detection networks

Two-stage detection networks have emerged as a principle approach in plant disease identification, effectively integrating region proposal mechanisms with classification tasks to enhance detection accuracy. The fundamental distinction between two-stage and one-stage networks lies in their processing pipelines: two-stage models first generate candidate regions potentially containing lesions and subsequently perform object detection within those regions, whereas one-stage models directly predict object locations and classes without a separate proposal stage (Wang et al., 2025).

Faster R-CNN, one of the most widely adopted two-stage object detection frameworks, has demonstrated high effectiveness in identifying plant diseases and pests. The network extracts a feature map from the input image using a convolutional backbone. Subsequently, a region proposal network (RPN) computes anchor box confidence scores to generate candidate regions, referred to as proposals. Following ROI pooling, the feature maps corresponding to these proposals are forwarded through subsequent network layers to refine detection, achieving accurate lesion localization and classification (Shao et al., 2019).

Building on this framework, Bari et al. (2021) employed Faster R-CNN for real-time detection of rice leaf diseases. Their model incorporated an improved RPN architecture capable of accurately localizing objects and generating robust candidate regions. The model was trained using both publicly available datasets and proprietary field images to enhance robustness. The study focused on three major rice leaf diseases: rice blast, brown spot, and hispa. The proposed approach achieved detection accuracies of 98.09% for rice blast, 98.85% for brown spot, and 99.17% for hispa, demonstrating high reliability across disease categories.

Gong and Zhang (2023) applied the Faster R-CNN framework to detect apple leaf diseases and compared its performance with that of YOLOv3. Their findings indicated that Faster R-CNN outperformed YOLOv3 in detection accuracy, primarily owing to its region proposal mechanism, which enables precise localization critical for capturing complex leaf disease patterns. Alruwaili et al. (2022) introduced a modified Faster R-CNN variant, termed RTFRCNN, designed for real-time detection of tomato leaf diseases. The model efficiently processed both static images and video streams, achieving an accuracy of 97.42%, surpassing AlexNet (96.32%) and conventional CNN models (92.21%) while requiring less computational power.

Similarly, Ozguven and Adem (2019) employed the Faster R-CNN framework to detect sugar beet leaf spot disease. Their model achieved a detection accuracy of 95.48%, effectively handling the natural variation in leaf morphology and confirming the suitability of DL-based approaches for conventional crops such as sugar beet. Mu et al. (2022) proposed a Faster R-CNN model with a feature pyramid network (FPN) backbone for detecting weed seedlings in agricultural fields. The model achieved an accuracy exceeding 95%, demonstrating the adaptability of the Faster R-CNN architecture for both disease detection and weed–crop discrimination under complex field conditions.

A summary of these studies is provided in Table 5.

**Figure 6:**
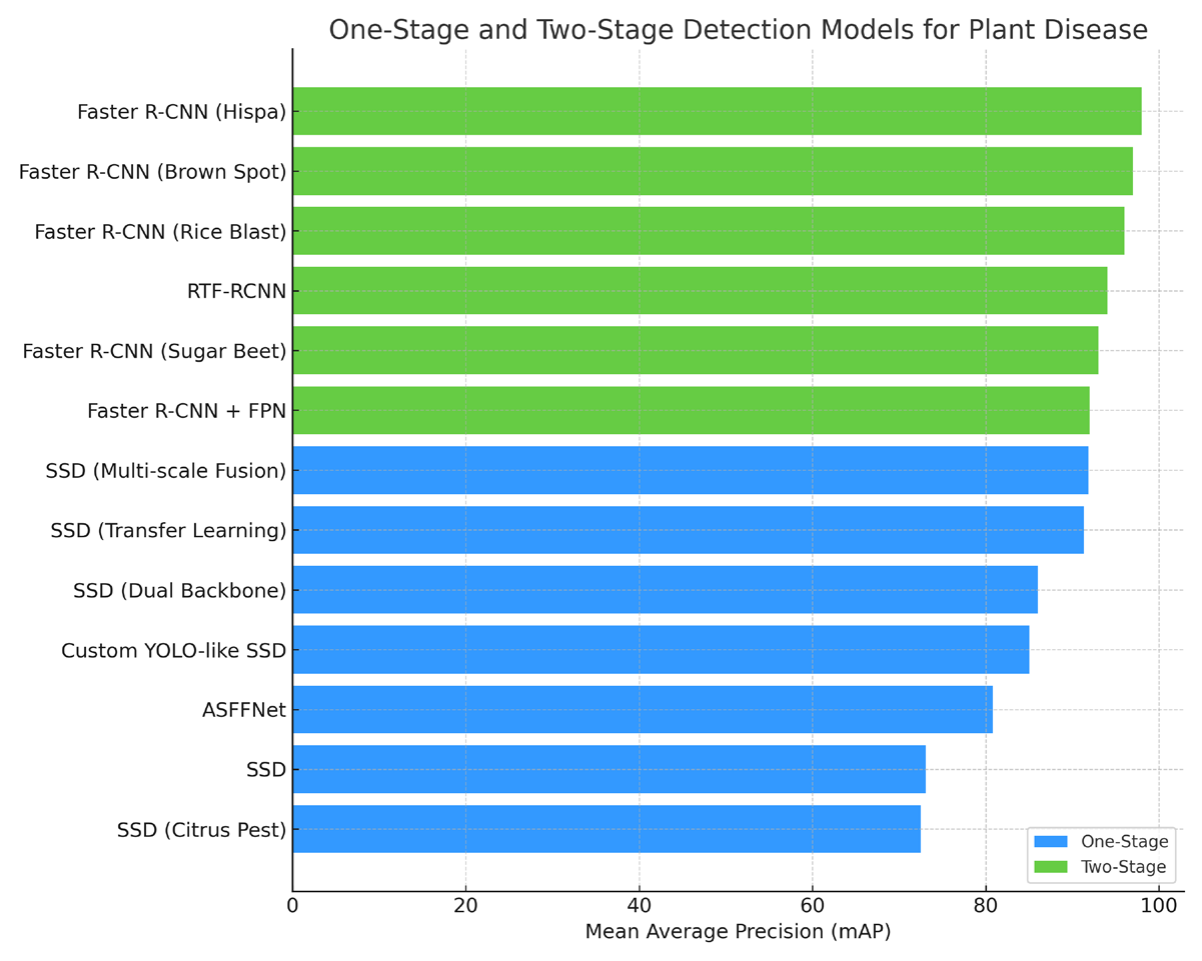
Deep learning models for plant disease detection categorized into one-stage and two-stage frameworks.

Figure 6 presents an overview of deep learning-based models for plant disease detection, systematically categorized into one-stage and two-stage architectures.

### 3.4. Image segmentation

Accurate detection of diseased regions on plant leaves is essential for reliable diagnosis and severity assessment. Object detection methods such as Faster R-CNN provide bounding boxes around affected areas but often lack the pixel-level precision required for detailed pathological analysis. Consequently, image segmentation techniques have gained increasing importance in plant disease analysis, as they enable precise isolation of infected leaf regions from healthy tissue.

In recent years, DL-based segmentation architectures such as U-Net, Mask R-CNN, fully convolutional networks (FCNs), SegNet, and DeepLabv3+ have been extensively adopted in agricultural research. These architectures offer the advantage of producing pixel-level segmentation masks that delineate infected regions, thereby supporting tasks such as monitoring disease progression, quantifying severity, and enabling precise treatment application. Furthermore, integrating segmentation with classification frameworks enhances the robustness and reliability of automated plant disease detection in real-world agricultural settings.

Mzoughi and Yahiaoui (2023) addressed the challenges of plant disease recognition using image-based DL approaches. Unlike conventional approaches that jointly classify disease–species pairs, they proposed a novel framework capable of identifying plant diseases independently of the host leaf species. This design enables the recognition of previously unseen plant species exhibiting known disease patterns. Moreover, instead of relying on entire leaf images, their method focuses on local symptomatic features to reduce contextual bias and enhance model generalization. The authors developed a hybrid system that integrates DL-based semantic segmentation and classification networks to extract infected regions and accurately identify corresponding diseases. Extensive experiments conducted on the PlantVillage dataset validated the effectiveness of utilizing local disease symptoms for classification. The proposed approach also achieved notable improvements on more complex datasets, such as IPM and BING, which include leaves captured under uncontrolled environmental conditions.

Xia et al. (2021) investigated DL–based image segmentation methods combined with unmanned aerial vehicle (UAV) imagery to detect pine wilt disease (PWD), a major ecological threat to pine forests. Using fixed-wing UAVs, they collected aerial imagery over pine forests in Laoshan, Qingdao, China, and complemented these data with ground-based surveys to obtain additional contextual information. A dataset comprising 2352 annotated samples of infected pine trees under varying background conditions was assembled. The study evaluated the performance of several state-of-the-art semantic segmentation architectures, including fully convolutional networks (FCN), DeepLabv3+, and PSPNet. Among the tested loss functions, focal loss outperformed Dice loss, improving the average intersection over union (IoU) from 0.656 to 0.701. DeepLabv3+ achieved the best segmentation performance, with an IoU of 0.720 and an F1-score of 0.832. The superior performance of the model was attributed to the atrous spatial pyramid pooling module and the encoder–decoder structure, which effectively captured multiscale contextual and spatial information. Notably, increasing the depth of backbone networks did not enhance segmentation performance, as neither ResNet34 nor ResNet50 proved optimal across models.

In another study, Zhang and Zhang (2023) proposed an improved U-Net architecture, termed MU-Net, to address the challenges of segmenting diseased leaf images characterized by irregular shapes, varying sizes, complex textures, and noisy backgrounds. To enhance segmentation performance, MU-Net incorporates residual blocks (ResBlocks) to mitigate gradient vanishing and explosion issues, while replacing conventional skip connections with residual paths (ResPaths) to improve feature transformation between the encoder and decoder branches. The integration of ResBlocks and ResPaths increases the network’s depth and representational capacity. Experimental evaluations on a dataset of diseased leaf images demonstrated that MU-Net achieved superior segmentation accuracy and computational efficiency compared to conventional approaches.

Wang et al. (2023) introduced a novel segmentation architecture termed MFBP-UNet. The model incorporates a multiscale feature extraction (MFE) module to enrich both detailed and semantic feature representations, along with a BATok-MLP component that employs tokenized multilayer perceptrons and dynamic sparse attention to effectively balance local and global feature extraction. In addition, a diffusion-based data augmentation strategy was employed to enhance the model’s robustness and training stability. Experimental evaluations demonstrated that MFBP-UNet outperformed existing segmentation models, achieving substantial improvements over the baseline U-Net across multiple performance metrics: mean intersection over union (mIoU) = 86.15%, mean precision (mP) = 93.53%, mean pixel accuracy (mPA) = 90.89%, and Dice coefficient = 0.922.

Similarly, Kaur et al. (2024) developed a hybrid DL model, termed Hybrid-DSCNN, to segment and detect tomato leaf diseases using the PlantVillage dataset. The dataset was systematically annotated, enhanced, and augmented to improve training efficiency. The Hybrid-DSCNN integrates pretrained U-Net and SegNet architectures with instance segmentation mechanisms to enhance object detection performance. The model enables semantic segmentation of both single and multiple disease types, allowing precise identification and classification. Comparative evaluations against modified U-Net, M-SegNet, and U-SegNet models demonstrated that Hybrid-DSCNN achieved superior performance across multiple evaluation metrics, including accuracy, precision, recall, intersection over union (IoU), and mean IoU (mIoU). A total of 1004 images were processed, yielding an accuracy of 98.24%, surpassing the performance of all competing models.

### 3.5. Real-world deployment and Edge AI

Although DL models have demonstrated high performance in experimental evaluations of plant disease classification, their deployment in real-world agricultural environments faces practical challenges related to computational resources, inference speed, connectivity, and power consumption. Edge computing and lightweight DL architectures have been proposed to address these challenges, enabling real-time, on-site diagnosis using resource-constrained devices such as mobile phones and embedded systems.

MobileNet, EfficientNet, and their respective variants have emerged as preferred architectures for deployment on such devices owing to their computational efficiency and compact design. Depthwise separable convolutions, as implemented in MobileNetV2, significantly reduce model complexity and computational cost while maintaining competitive accuracy. Chen et al. (2021) developed a MobileNetV2-based model enhanced with channel-wise attention and dual-stage transfer learning, achieving 99.67% accuracy on a public plant disease dataset and demonstrating excellent suitability for mobile deployment. Similarly, Hassan et al. (2021) demonstrated real-time inference capabilities using MobileNetV2 and EfficientNetB0, achieving up to 99.56% accuracy across 38 disease categories and 14 plant species.

Beyond accuracy optimization, model compression techniques—such as quantization, pruning, and knowledge distillation—have attracted significant attention for reducing computational complexity and memory requirements. Khan et al. (2023b) applied quantization to a MobileNetV3-Small architecture, reducing the parameter count to 0.93 million while maintaining a classification accuracy of 99.50%. The optimized model, exported in open neural network exchange (ONNX) format, supports deployment across diverse edge platforms, including mobile devices. Guan et al. (2023) proposed a custom lightweight architecture, Dise-Efficient, derived from EfficientNetV2, which achieved 99.80% accuracy on the PlantVillage dataset.

Karim et al. (2024) designed a modified MobileNetV3-Large model for real-time grape leaf disease detection and deployed it on an NVIDIA Jetson Nano edge computing device. The system achieved 99.66% training accuracy and 99.42% testing accuracy and incorporated gradient-weighted class activation mapping (Grad-CAM) for visual interpretability, thereby enhancing model transparency and practical usability under field conditions.

## 4. Data and datasets

### 4.1. Publicly available datasets

**Figure 7:**
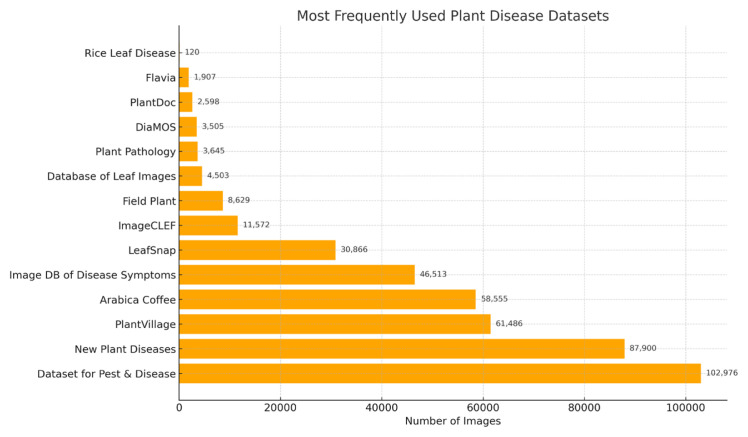
Proportional distribution of commonly used plant disease datasets.

Training DL models requires large and diverse datasets, as both the quantity and variability of data significantly influence the effectiveness of the learning process. Robust datasets encompassing images from multiple plant species and disease categories enable DL models to accurately identify a wide range of leaf anomalies. Dataset diversity is essential for developing models that generalize effectively to real-world conditions and minimize overfitting to specific environmental or imaging conditions. This section provides an overview of the most widely used plant disease datasets reported in the literature. Table 6 summarizes publicly available repositories, while Figure 7 illustrates the distribution of commonly used datasets according to their image counts.

#### 4.1.1. PlantVillage dataset

The PlantVillage dataset^1^, introduced within the domains of agriculture and computer vision, has gained widespread recognition. It has become a cornerstone for developing and training DL-based models targeting key tasks such as plant disease identification and severity estimation, comprising an extensive collection of 61,486 images.

This extensive repository distinguishes itself as one of the largest and most comprehensive datasets available in the field. The dataset includes 14 crop species and 39 distinct disease classes, effectively capturing the variability and complexity of plant health conditions.

The PlantVillage dataset is distinguished by its diverse sources, incorporating contributions from both researchers and farmers. This multisource approach ensures that the dataset reflects the real-world complexity of plant diseases by capturing variations in image quality, disease stages, and environmental conditions. Each image in the repository is meticulously labeled and annotated with identifiers specifying the corresponding plant disease. Accurate labeling is fundamental in supervised ML, providing the foundation for training DL models to identify and classify plant diseases with high precision.

#### 4.1.2. Leaf Image database

The leaf image database^2^ consists of 4503 unique images that have been systematically curated and categorized for research purposes. Of these, 2278 images depict healthy leaves, whereas 2225 correspond to diseased samples. The dataset includes 22 distinct categories representing various plant species and their respective health conditions. All images were captured under controlled laboratory conditions at Shri Mata Vaishno Devi College, Katra, India. The dataset is organized into two primary categories: healthy and diseased. Each image was further grouped according to plant species, labeled sequentially from P0 to P11. Subsequently, the entire dataset was subdivided into 22 subject categories, numbered from 0000 to 0022. Categories 0000–0011 correspond to healthy leaves, while 0012–0022 represent diseased samples. This structured categorization provides researchers and practitioners with a comprehensive resource for analyzing leaf images across diverse species and health conditions.

#### 4.1.3. LeafSnap dataset

The LeafSnap^3^ dataset consists of 30,866 images representing various leaf types. Of these, 23,147 are high-quality images captured under controlled conditions using both backlight and reflected-light techniques. The remaining 7719 images were collected in field conditions using mobile devices. These field images depict leaves in their natural, unpressed state. Each image in this dataset is meticulously labeled with the corresponding tree species to which the leaf belongs. The dataset encompasses 185 distinct leaf classes, providing extensive species-level representation. Additionally, the dataset provides segmentation masks for leaf images. However, the segmentation process occasionally encounters errors, producing fully black segmentation masks in certain instances.

#### 4.1.4. ImageCLEF dataset

The ImageCLEF^4^ dataset focuses on 126 tree species native to the French Mediterranean region for plant identification research. It consists of 11,572 images categorized into three types: scans (57%), scan-like photographs (24%), and natural field images (19%). The training set contains 8422 images, including 4870 scans, 1819 scan-like photographs, and 1733 natural images, each provided with XML annotation files. The test set comprises 3150 images—1760 scans, 907 scan-like photographs, and 483 natural images—all supplied with XML annotation files.

#### 4.1.5. PlantDoc dataset

The PlantDoc dataset (Singh et al., 2020) contains 2598 images representing 13 plant species and 17 distinct disease classes. Its development required approximately 300 human hours of meticulous annotation. To evaluate the dataset’s effectiveness, three DL models were trained for plant disease classification tasks. The results indicated that using this dataset improved classification accuracy by up to 31%.

#### 4.1.6. Plant disease symptom image database

The image database of plant disease symptoms (PDDB) (Garcia Arnal Barbedo et al., 2018) contains 2326 images depicting 171 diseases and disorders across 21 plant species. Although the original dataset is sizable, it remains insufficient for training high-capacity DL models. To overcome this limitation, each image was subdivided according to defined criteria, expanding the dataset to 46,513 samples. Both the original PDDB and its expanded version (XDB) are publicly available for academic research.

#### 4.1.7. Rice leaf disease dataset

The rice leaf disease dataset (Shah et al., 2019) serves as a valuable resource for researchers and practitioners specializing in rice pathology and agriculture. It contains 120 JPEG images of rice leaves affected by three major diseases: leaf spot, brown spot, and bacterial leaf blight. Each disease class comprises 40 images, facilitating the development and evaluation of ML models for disease classification and identification. Accurate diagnosis of rice leaf diseases is essential for effective management, helping farmers and researchers prevent outbreaks and maintain healthy crops. This dataset provides a foundation for developing automated disease detection systems aimed at improving rice productivity and plant health, making it a valuable asset for the research community.

#### 4.1.8. Flavia leaf dataset

The Flavia dataset (Wu et al., 2007) is one of the most well-known benchmark datasets for leaf recognition. It contains 1907 leaf images representing 33 distinct plant species. Each image has a resolution of 1200 × 1600 pixels and is freely available for research use.

#### 4.1.9. DiaMOS plant dataset

The DiaMOS plant dataset (Fenu and Malloci, 2021) is a comprehensive resource containing images captured throughout the full growing season of pear trees, from February to July. The dataset aims to provide representative samples covering the primary developmental and cultural stages of the plant. It contains 3505 images in total, including 499 fruit images and 3006 leaf images, making it suitable for ML and DL applications in classification and detection.

#### 4.1.10. FieldPlant dataset

The FieldPlant dataset (Moupojou et al., 2023) consists of 8629 annotated field images of leaves collected from plantations in Cameroon. It focuses on detecting and identifying diseases affecting three major tropical crops: corn, cassava, and tomato. Notably, FieldPlant is the first publicly available dataset to include annotated cassava images for plant disease detection tasks. This dataset provides a valuable resource for training efficient DL models for plant disease detection under real-world field conditions, supporting the advancement of object detection approaches.

#### 4.1.11. New plant disease dataset

The new plant disease dataset^5^ contains approximately 87,900 RGB images of healthy and diseased crop leaves, categorized into 38 distinct classes. The dataset is divided into training and validation subsets using an 80/20 split, while preserving the original directory structure. Additionally, a separate directory containing 33 test images is provided for prediction and evaluation purposes.

#### 4.1.12. CCMT dataset

The CCMT dataset (Mensah et al., 2023) was specifically developed for detecting crop pests and diseases. It contains images collected from local farms in Ghana and is available in two formats: raw and augmented. The raw dataset comprises 24,881 images categorized as follows: 6549 cashew, 7508 cassava, 5389 maize, and 5435 tomato samples. The augmented dataset, divided into training and testing subsets, contains 102,976 images across 22 categories: 25,811 cashew, 26,330 cassava, 23,657 maize, and 27,178 tomato samples. All images were anonymized, validated by expert plant virologists, and made publicly available to the research community.

#### 4.1.13. Plant pathology dataset

The plant pathology dataset (Thapa et al., 2020) contains 3645 images depicting various symptoms of apple foliar diseases. These images were collected during the 2019 growing season from commercially grown apple cultivars in an unsprayed orchard at Cornell AgriTech, Geneva, NY, USA. Among the 3645 RGB images, 1200 depict apple scab, 1399 show cedar apple rust, 187 display complex symptoms involving multiple diseases on the same leaf, and 865 correspond to healthy samples. The images were captured in field conditions using smartphones under varying illumination, viewing angles, surface textures, and noise levels. The dataset was manually annotated into four categories—cedar apple rust, apple scab, multiple diseases, and healthy leaves—with all annotations verified by an expert plant pathologist.

#### 4.1.14. Citrus fruit and leaf dataset

The citrus dataset (Rauf et al., 2019) contains 759 images of healthy and diseased citrus fruits and leaves. The images were manually captured using a DSLR camera under the supervision of domain experts to ensure labeling accuracy. The diseased samples were categorized into five groups: black spot, canker, scab, greening, and melanose. All images were resized to 256 × 256 pixels with a resolution of 72 dpi. Citrus fruit images were captured directly from plants in their natural environment, whereas leaf images were acquired under controlled laboratory conditions against a uniform gray background.

#### 4.1.15. BRACOL coffee leaf dataset

The BRACOL dataset (Esgario et al., 2019) comprises images of Arabica coffee leaves specifically developed for the identification and quantification of coffee diseases and pests. It includes 2147 images depicting leaves affected by biotic stresses such as leaf miner, coffee leaf rust, brown leaf spot, and Cercospora leaf spot. These images were collected throughout the year in Santa Maria, Marechal Floriano—a mountainous region in Espírito Santo, Brazil. Captured using five different smartphones, the images focus on the abaxial (lower) leaf surface, with samples placed on a white background under partially controlled lighting conditions. The data collection process was intentionally diversified to enhance dataset variability, and annotation was performed with the assistance of an expert in plant biotic stress identification.

#### 4.1.16. Apple leaf disease detection dataset

The apple leaf disease detection (ALDD) dataset (Jiang et al., 2019) contains 2029 images of apple leaves affected by five distinct diseases. The images were collected under diverse weather conditions, introducing natural variability and complex backgrounds that may challenge certain experiments. Following annotation and data augmentation, the dataset was expanded to 26,377 images, improving its suitability for DL model training.

#### 4.1.17. Plant disease recognition dataset

The open-access plant disease recognition dataset^6^ comprises 1530 images categorized into three classes: healthy, powdery, and rust. These categories represent different plant health conditions: healthy indicates disease-free leaves, whereas powdery and rust denote leaves affected by specific fungal infections. The dataset is divided into training, validation, and testing subsets, which are essential for developing and evaluating ML models. Automated recognition and diagnosis of plant diseases through image analysis are critical in agriculture and horticulture, enabling early intervention, crop preservation, and improved productivity. This dataset serves as a valuable benchmark for researchers and ML practitioners developing automated plant disease recognition systems.

#### 4.1.18. Kashmiri apple disease dataset

The dataset was collected from orchards in the Kashmir Valley for educational and research purposes and is publicly available on the Kaggle platform. It comprises approximately 419 images depicting both healthy and diseased leaves. Data collection took place during May, June, and July, coinciding with the peak season of plant disease prevalence. All images were manually captured using digital cameras and mobile phones from various brands (Sharma et al., 2022).

#### 4.1.19. Groundnut leaf disease dataset

The groundnut leaf disease dataset (Aishwarya and Reddy, 2023) consists of digital photographs of groundnut leaves collected in the Koppal region of Karnataka, India, with the assistance of a plant pathologist under natural field conditions. The images are categorized into six classes based on leaf condition: healthy, early leaf spot, late leaf spot, nutritional deficiency, rust, and early rust. After preprocessing, the dataset contains 10,361 images organized into six folders: healthy leaves (1871), early leaf spot (1731), late leaf spot (1896), nutritional deficiency (1665), rust (1724), and early rust (1474). This comprehensive dataset serves as a valuable benchmark for training and validating ML and DL models in groundnut leaf disease classification and recognition.

#### 4.1.20. Arabica coffee leaf dataset

The Arabica coffee leaf datasets, JMuBEN and JMuBEN2, were collected under real-world field conditions at the Mutira coffee plantation in Kirinyaga County, Kenya, using a digital camera and guided by an expert plant pathologist. The JMuBEN dataset includes three compressed folders: one containing 7682 images of Cercospora, another with 8337 images of coffee rust, and a third with 6572 images of Phoma. Conversely, the JMuBEN2 dataset consists of two compressed folders: one with 16,979 images of leaf miner infestations and another with 18,985 images of healthy leaves. Combined, the two datasets contain 58,555 annotated images across five categories: Phoma, Cercospora, rust, healthy, and leaf miner. Together, these datasets provide a valuable benchmark for training and validating DL models designed to recognize and classify Arabica coffee leaf diseases (Jepkoech et al., 2021).

#### 4.1.21. Tobacco disease dataset

The tobacco plant disease dataset (Lin et al., 2022) comprises 2721 images of tobacco leaves captured under real-world field conditions. The dataset serves a dual purpose, supporting both disease classification and leaf detection tasks. The disease classification subset provides a diverse collection of images, enabling researchers and ML practitioners to develop models capable of accurately identifying and classifying multiple tobacco leaf diseases. It also supports leaf detection, which is essential for tracking and monitoring plant health and growth in agricultural applications. The combination of these two tasks makes the dataset particularly valuable for addressing challenges in tobacco disease management and ensuring the health and quality of tobacco crops.

#### 4.1.22. Robusta coffee leaf dataset

The RoCoLe dataset (Parraga-Alava et al., 2019) consists of 1560 images of Robusta coffee leaves captured under real-world field conditions at a single plantation using a smartphone camera. It includes images showing visible red mites and rust spots indicative of coffee leaf rust infection, as well as healthy leaves without visible symptoms. Each image is annotated with details regarding the leaf objects, their health status (healthy or diseased), and disease severity (the proportion of leaf area affected by spots). RoCoLe serves as a valuable benchmark for evaluating ML algorithms in image segmentation and classification tasks related to plant disease recognition.

#### 4.1.23. Black gram leaf disease dataset

The black gram plant leaf disease (BPLD) dataset (Talasila et al., 2022) focuses on *Vigna mungo* (commonly known as Urad), one of the most important pulse crops cultivated in India. The crop is severely affected by diseases such as anthracnose, leaf crinkle, powdery mildew, and yellow mosaic, which cause substantial yield losses among farmers. To facilitate early detection and classification, a dataset of 1000 images was created, comprising five classes: four disease categories and one healthy class. The images were captured using cameras and mobile phones under natural cultivation conditions in Nagayalanka, Krishna District, Andhra Pradesh, India. Agricultural experts assisted in labeling and processing the images. This dataset serves as a valuable resource for researchers applying image processing, ML, and DL techniques for the automated diagnosis and classification of black gram leaf diseases, thereby supporting farmers in disease management and yield improvement.

#### 4.1.24. Sunflower fruit and leaf dataset

This dataset consists of images of healthy and diseased sunflower leaves and flowers affected by downy mildew, gray mold, and leaf scar disorders. The images were manually captured between 25–29 November 2021, at the Bangladesh Agricultural Research Institute (BARI) demonstration farm in Gazipur, in collaboration with an agricultural domain expert. The data collection period coincided with the sunflower plants approaching full bloom—a stage when disease incidence was as its peak. The original dataset contains 467 images collected from the sunflower demonstration field. To increase dataset size and variability, data augmentation techniques were applied, expanding the total to 1668 images in the augmented version (Sara et al., 2022).

#### 4.1.25. Bangladeshi crop disease dataset

The new Bangladeshi crop disease dataset^7^ encompasses a wide variety of plant species, with a primary focus on four major crops: corn, potato, rice, and wheat. It contains 14 classes and 13,024 images, making it a valuable benchmark for plant disease recognition and classification research.

#### 4.1.26. Wheat disease dataset

This dataset comprises a modest yet representative subset of wheat disease images, totaling 999 samples. These images depict real-world wheat growth conditions and are categorized into five distinct classes: yellow rust, brown rust, septoria, mildew, and healthy leaves. It is important to note that this subset represents only a fraction of the full dataset, which contains 19,172 images across the same five classes. The smaller subset provides a practical starting point for researchers and ML practitioners to develop and test models for wheat disease recognition and classification. The full dataset, with its comprehensive diversity of images, serves as a more robust and extensive resource for addressing challenges in wheat disease detection and agricultural improvement (Long et al., 2023).

#### 4.1.27. New Zealand fungal and plant disease collection

The New Zealand fungal and plant disease collection (PDD) (Wilton, 2019) represents a major biodiversity repository encompassing a wide range of organism groups. Within its extensive holdings, fungi constitute the most represented group, with 106,016 samples. In addition, the collection includes diverse groups such as protozoa (2343), chromista (1261), plantae (119), bacteria (113), and a small number classified as *incertae sedis* (11). The PDD’s extensive diversity makes it an essential resource for researchers studying and preserving New Zealand’s unique fungal and plant biodiversity, including potentially novel species and their ecological interactions.

#### 4.1.28. Sugarcane leaf disease dataset

The manually collected sugarcane leaf disease dataset serves as an essential resource for researchers and agricultural practitioners. It contains 2569 images categorized into five distinct classes: healthy, mosaic, red rot, rust, and yellow disease. The images were captured using smartphones with varied configurations, ensuring diversity in the dataset. The variety of devices used for image capture reflects real-world field conditions, making this dataset robust for developing ML models and algorithms. Collected in Maharashtra, India, the dataset is region-specific, offering valuable insights into sugarcane diseases prevalent in that region. The balanced distribution of images across categories enhances its utility, making it an ideal resource for training and testing sugarcane disease detection models. With variations in image size and RGB format, the dataset realistically represents the challenges encountered in real-world applications, facilitating the development of accurate and adaptable DL-based disease detection solutions for sugarcane crops (Daphal and Koli, 2023).

#### 4.1.29. Tomato leaf dataset

The tomato leaf image collection comprises two distinct datasets, each derived from existing image repositories. The first dataset contains tomato leaf images extracted from the PlantVillage database, comprising 10 categories—nine disease classes and one healthy class. This substantial dataset includes 14,531 images, each depicting a single tomato leaf with its natural background. To streamline and refine the dataset, certain categories were excluded, resulting in a more focused and balanced collection. All images were resized from 256 × 256 pixels to 227 × 227 pixels to optimize them for further analysis and model development. The dataset is organized for practical evaluation using a five-fold cross-validation approach, ensuring robust testing and validation of ML models on this resource. This dataset serves as a critical benchmark for developing and evaluating models aimed at detecting tomato leaf diseases and supporting related research efforts (Huang and Chang, 2020).

### 4.2. Data collection and acquisition

**Figure 8:**
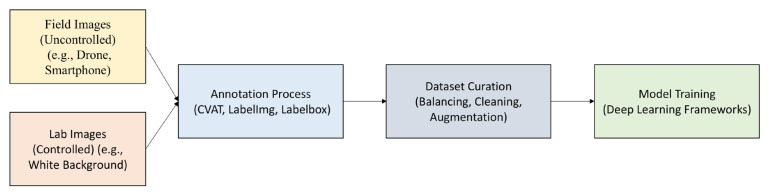
Overview of the plant disease data acquisition pipeline.

The performance and reliability of DL-based plant disease detection systems are highly dependent on the quality, diversity, and quantity of data used during both training and evaluation. Data collection represents the foundational step in developing robust DL models and includes the acquisition, annotation, and curation of datasets (Ferentinos, 2018). Figure 8 illustrates a conceptual overview of the data acquisition workflow for plant disease detection. The process begins with capturing images under both uncontrolled field environments and controlled laboratory conditions. These images are subsequently annotated using dedicated labeling tools such as CVAT, LabelImg, and Labelbox. Following annotation, the data undergoes a curation phase—comprising cleaning, classification, and expansion—to ensure model trainability and consistency. Finally, the curated dataset is used to train DL models for disease recognition and classification tasks.

Image acquisition for plant disease detection generally occurs in two primary contexts: under field conditions and in controlled laboratory environments. Field-acquired data exhibit natural variations due to factors such as inconsistent lighting, occlusions, complex backgrounds, and ambient environmental noise. In contrast, laboratory-acquired images benefit from uniform lighting and controlled backgrounds, resulting in cleaner datasets that may, however, lack real-world variability. Both approaches present distinct advantages and limitations, and an effective DL model must be capable of generalizing across these variations (Mohanty et al., 2016).

Another major challenge lies in the accurate annotation of disease symptoms, which frequently requires domain expertise due to the visual similarity among different disease manifestations. The annotation process may involve simple class labels, bounding boxes for object detection, or pixel-level segmentation masks for semantic segmentation tasks. Tools such as LabelImg (Mathew and Mahesh, 2022), CVAT (Yue et al., 2025), and Labelbox (Chaudhari et al., 2023) facilitate this process; however, the required human effort remains considerable.

Beyond annotation quality, dataset imbalance remains a persistent and critical issue. Certain disease classes are well-represented, whereas others suffer from limited sample availability, potentially distorting the model’s learning process. Although data augmentation techniques are widely employed to artificially enhance sample diversity, the collection of geographically diverse, real, and balanced datasets remains an essential priority for future research (Ojo and Zahid, 2023).

### 4.3. Data preprocessing pipeline

**Figure 9:**
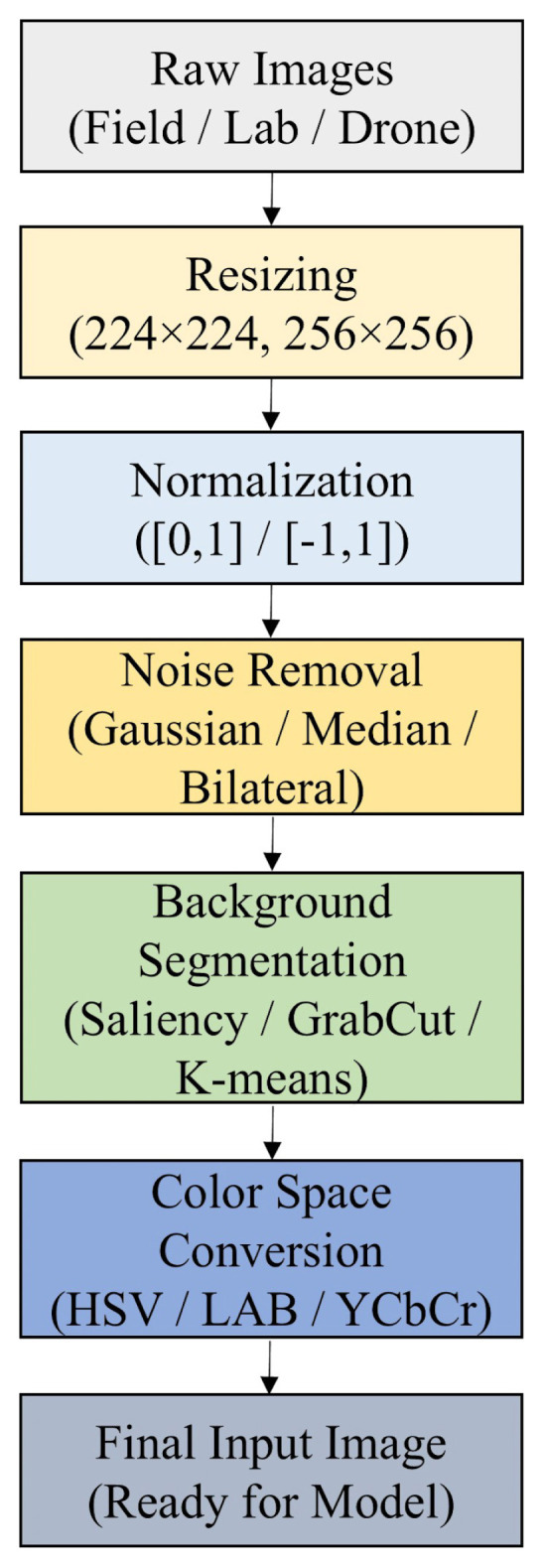
Workflow of data preprocessing for plant disease detection.

Data preprocessing constitutes a vital stage in the DL workflow for plant disease detection, directly influencing model accuracy, generalization capability, and robustness. Although raw images capture the real-world complexity of plant diseases, they frequently contain noise, inconsistencies, and irrelevant visual details that may hinder the learning process (Ojo and Zahid, 2023). Effective preprocessing transforms raw data into a standardized and model-compatible format, ensuring consistency while improving the signal-to-noise ratio. Figure 9 presents a structured and visually enhanced workflow outlining the data preprocessing pipeline for DL-based plant disease detection. The process begins with the collection of raw images from diverse sources, including field environments, laboratory setups, and unmanned aerial vehicle (UAV) systems. These images undergo several preprocessing transformations, including resizing to match model input dimensions, pixel normalization to stabilize training, and noise filtering to eliminate irrelevant visual artifacts. Subsequently, background segmentation is applied to isolate plant regions, often followed by conversion into perceptually enhanced color spaces such as hue–saturation–value (HSV) or lightness–A–B color spaces (LAB) to improve feature representation. The output of this preprocessing pipeline serves as the standardized input for DL models, exerting a significant influence on both classification accuracy and generalization performance.

Images collected from multiple acquisition devices—including smartphones, DSLR cameras, and UAVs—typically vary in resolution, format, and aspect ratio (Shafik et al., 2023). DL architectures, such as VGG16, ResNet, and MobileNet require fixed input dimensions; therefore, image resizing constitutes a necessary preprocessing step. Commonly used input sizes include 224 × 224, 227 × 227, and 256 × 256 pixels, providing a practical balance between spatial detail preservation and computational efficiency.

Normalizing pixel intensity values to standardized ranges, such as [0,1] or [−1,1], ensures numerical stability throughout the training process. This normalization process accelerates convergence by aligning the input data distribution with the initialization and learning rate configurations of neural networks (NNs) (Gursoy and Kaya, 2024). Field-acquired images often contain nonleaf elements—such as soil, sky, or surrounding vegetation—that act as irrelevant background clutter. Such background elements can misdirect the model’s attention and introduce undesired correlations during feature learning. To mitigate this issue, several studies have implemented denoising techniques such as bilateral filtering, Gaussian blurring, and median filtering, which effectively suppress image noise while preserving edge information (Bhujade et al., 2024).

Background segmentation—where the diseased leaf is isolated from the surrounding scene—has proven beneficial for improving detection and classification accuracy. Barbedo (2018) proposed a saliency-based segmentation approach, which significantly enhanced classification accuracy for tomato leaf disease detection. Other commonly employed segmentation methods include thresholding in the HSV color space, k-means clustering, and GrabCut, all of which have been shown to improve model performance in recognition and segmentation tasks. Color transformation represents another essential component of preprocessing in plant disease detection, as disease symptoms—such as yellowing, necrosis, and rust—manifest through distinct chromatic variations. Although the RGB color space is commonly used, it is not always the most optimal representation for capturing subtle disease symptoms. Converting images into alternative color spaces such as HSV, LAB, or YCbCr enhances model sensitivity to hue and saturation variations, thereby enabling more accurate color-based disease classification (Trivedi et al., 2022).

#### 4.3.1. Data augmentation techniques

**Figure 10:**
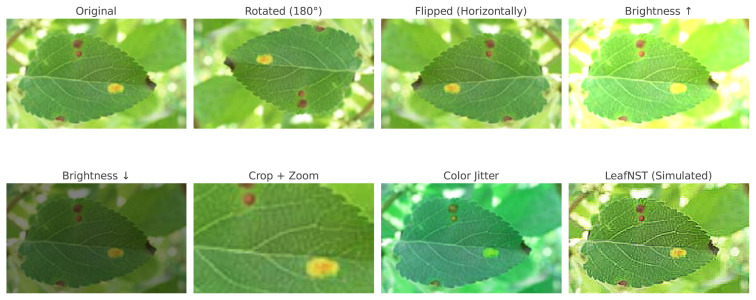
Examples of data augmentation applied to a single leaf image. Augmentations include rotation, flipping, brightness adjustment, cropping, color jittering, and simulated neural style transfer (LeafNST).

Data augmentation plays a vital role in training DL models for plant disease detection, particularly in mitigating class imbalance, reducing overfitting, and enhancing model generalization. Since many plant disease datasets contain a limited number of samples—especially for rare or region-specific diseases—augmentation techniques are essential for achieving robust and reliable model performance (Ngugi et al., 2024). Data augmentation artificially expands the diversity of the training set by applying geometric and photometric transformations to existing images. Common operations include horizontal and vertical flipping, rotation (e.g., 90°, 180°), random cropping, zooming, shearing, brightness and contrast adjustment, and color shifting. Figure 10 illustrates several augmentation techniques applied to a single diseased leaf image.

Numerous studies have employed these techniques to enhance the performance of DL-based plant disease detection models. Min et al. (2023) proposed a data augmentation strategy based on image-to-image translation with attention mechanisms, generating realistic synthetic images of diseased leaves that improved classification accuracy. Khare et al. (2024) introduced LeafNST, a neural style transfer (NST)-based augmentation framework that synthesizes object-level textures to address dataset imbalance in plant disease images. This method generated diverse and highly realistic synthetic images, significantly improving both model generalization and accuracy. Furthermore, Muhammad et al. (2023) explored diffusion-based augmentation and demonstrated its superiority over traditional GAN-based methods in classifying coffee leaf diseases. Collectively, these studies underscore the effectiveness of both conventional and generative augmentation techniques in improving the robustness and predictive accuracy of DL models for plant disease detection.

## 5. Evaluation metrics for deep learning models

Various evaluation metrics have been widely adopted in the literature to assess the performance of DL models applied to plant disease classification, detection, and segmentation tasks. This section outlines the most commonly used evaluation metrics suitable for assessing classification, detection, and segmentation performance.

### 5.1. Accuracy

Accuracy measures the overall correctness of model predictions and is defined as:


(1) $$Accuracy=\frac{\text{TP}+\text{TN}}{TP+TN+FP+FN}$$

### 5.2. Precision, recall, and F1-score

Precision quantifies the proportion of correctly predicted positive cases among all instances predicted as positive:


(2) $$Precision=\frac{\text{TP}}{TP+FP}$$

Recall (also referred to as sensitivity) measures the model’s ability to correctly identify all actual positive instances:


(3) $$Recall=\frac{\text{TP}}{TP+FN}$$

The F1-score represents the harmonic mean of precision and recall, balancing the trade-off between the two metrics:


(4) $$F1-Score=2\cdot \frac{\text{Precision}\cdot \text{Recall}}{Precision+Recall}$$

### 5.3. Specificity

Specificity reflects the model’s capacity to correctly identify negative cases and is defined as:


(5) $$Specificity=\frac{\text{TN}}{TN+FP}$$

### 5.4. Confusion matrix

The confusion matrix provides a detailed representation of the model’s prediction outcomes for each class and is widely employed in evaluating multiclass classification performance.

### 5.5. ROC curve and AUC

The receiver operating characteristic (ROC) curve plots the true positive rate (TPR) against the false positive rate (FPR) across multiple threshold settings. The area under the curve (AUC) quantifies the model’s overall discriminative capability across all possible classification thresholds.


(6) $$FPR=\frac{FP}{FP+TN},TPR=\frac{TP}{TP+FN}$$

### 5.6. Intersection over union

The intersection over union (IoU) is a standard metric in object detection and image segmentation that quantifies the spatial overlap between predicted and ground-truth bounding boxes or segmented regions:


(7) $$IoU=\frac{\text{Area of Overlap}}{Area\;of\;Union}$$

### 5.7. Mean average precision

The mean average precision (mAP) represents the mean of the average precision (AP) values computed across multiple classes and IoU thresholds, serving as a key evaluation metric in object detection tasks:


(8) $$mAP=\frac{1}{N}\Sigma_{i=1}^{N}AP_{i}$$

### 5.8. Rank biased overlap

In metalearning frameworks, rank biased overlap (RBO) measures the similarity between ranked lists of model recommendations, where values range from 0 (no overlap) to 1 (perfect agreement).

### 5.9. Training and inference efficiency

Several studies additionally report computational efficiency metrics—such as training time, inference latency, and model size—to evaluate the feasibility of real-time and edge-based applications.

Collectively, these metrics provide a comprehensive framework for evaluating model effectiveness, particularly in addressing real-world agricultural challenges such as class imbalance, multiclass classification, and spatial localization accuracy.

## 6. Discussion

Deep learning (DL) models employed for plant disease diagnosis encompass diverse architectures, learning paradigms, visualization strategies, and segmentation and detection techniques, alongside practical considerations for real-world deployment. The accumulated evidence highlights substantial advancements in this domain while simultaneously underscoring key trends, strengths, limitations, and potential directions for further research. Although the field of DL-based plant disease diagnosis has expanded considerably, it remains fragmented in terms of model selection, evaluation methodologies, and deployment feasibility.

Numerous high-performing architectures, such as DenseNet201, InceptionResNetV2, and ResNet-50, have demonstrated outstanding classification accuracy on benchmark datasets like PlantVillage. However, their substantial computational demands pose major challenges for real-time inference and mobile deployment. In contrast, lightweight architectures such as MobileNetV2 and EfficientNetB0 (Chen et al., 2021; Hassan et al., 2021) demonstrate that marginal sacrifices in accuracy can significantly enhance deployability on edge devices. Nevertheless, few studies have systematically compared these models under consistent experimental conditions, raising questions about the acceptable trade-off between accuracy and computational efficiency. For instance, the notable accuracy achieved by a modified MobileNetV3 model deployed on Jetson Nano (Karim et al., 2024) effectively illustrates this trade-off between accuracy and efficiency..

Segmentation is often regarded as an alternative to classification due to its capability to localize and quantify disease severity. However, not all applications demand pixel-level precision; for example, classification may suffice in commercial crop sorting, whereas segmentation becomes essential in precision spraying. Studies such as Mzoughi and Yahiaoui (2023) demonstrate the potential of segmentation; however, few explicitly quantify how this added granularity translates into tangible practical outcomes. Moreover, segmentation-based models often require pixel-wise annotations—a frequently overlooked bottleneck that significantly constrains scalability and large-scale deployment.

Visualization techniques such as gradient-weighted class activation mapping (Grad-CAM) and saliency maps have become standard tools for evaluating DL models (Karim et al., 2024). However, many implementations rely solely on visual inspection, without systematically assessing the impact of explainability on user trust, model debugging, or agronomic decision-making. Despite their appeal, current explainable AI (XAI) methods lack standardized interpretability metrics, making them largely qualitative rather than operationally actionable. Therefore, there is a pressing need for usability studies to assess whether farmers and domain experts perceive these visualizations as interpretable and practically valuable.

Generative adversarial network (GAN)-based image synthesis, as explored by Muhammad et al. (2023) and Abbas et al. (2021), is frequently proposed as a solution to the data scarcity problem in plant disease classification. However, few studies have rigorously evaluated whether GAN-generated images faithfully preserve the pathological features necessary for reliable classification. Without extensive testing on real-world and unseen samples, models trained with GAN-augmented data risk overfitting to artificial artifacts rather than genuine disease characteristics. Although GANs offer significant potential, they cannot replace the need for diverse, well-annotated datasets collected under real-world field conditions.

Recent studies have begun incorporating transformer-based attention mechanisms and vision transformer (ViT)-style architectures (Alirezazadeh et al., 2023; Tabbakh and Barpanda, 2023). However, transformer architectures are often data-hungry and tend to be overparameterized for relatively simple plant disease imagery. It remains unclear whether these complex architectures provide a tangible advantage over CNNs in plant disease detection or if their adoption merely reflects broader trends in computer vision research. Future research should evaluate transformer performance on multimodal or geographically diverse datasets, where their capacity for modeling complex spatial and contextual relationships may be better demonstrated. Field conditions introduce challenges such as noise, occlusion, uneven illumination, and device variability—factors that are largely absent in controlled laboratory datasets. Although several studies have simulated real-world deployment on devices such as Jetson Nano or smartphones (Karim et al., 2024), few have rigorously validated their models under diverse and uncontrolled field environments. Moreover, aspects such as inference latency, power consumption, and user interface design are rarely subjected to systematic evaluation. Without comprehensive empirical assessment, deployability remains an assumption rather than a demonstrated capability.

Chug et al. (2023) reported that images containing multiple or overlapping leaves limit the applicability and generalization capability of their model. The study also identified limited dataset size as a major constraint (Min et al., 2023), potentially affecting the model’s accuracy and robustness. Notably, the current research did not investigate the segmentation of individual leaves from clusters, a preprocessing step that could potentially enhance classification accuracy.

Another major challenge in plant disease detection is the high similarity of geometric and morphological features among different disease classes (Bari et al., 2021). Disease identification and classification are further complicated by low-contrast image information, chromatic similarities between healthy and infected regions, and the presence of image noise. Model performance may also degrade due to noise, blur, and other distortions present in input samples (Albattah et al., 2022).

Min et al. (2023) noted that the vanilla CycleGAN architecture exhibits limitations in accurately capturing the distinct textural characteristics of target diseases. They further expressed concern regarding shape preservation in input leaf images due to indiscriminate background transformations during synthesis. The authors also highlighted that collecting diverse disease samples may exacerbate class imbalance issues within the dataset.

Lee and Yun (2023) reported that model performance varies depending on the types of environmental variables considered and the duration of environmental monitoring, emphasizing the importance of parameter optimization. Developing a comprehensive and well-structured database is essential before these models can be effectively deployed across diverse agricultural scenarios. Model usability and predictive performance could be enhanced by integrating additional sensors to capture a broader spectrum of environmental variables, reflecting current limitations in data acquisition. The study did not account for nonenvironmental factors that may influence crop disease prediction, potentially limiting the model’s overall accuracy.

Despite achieving high accuracy, few studies have addressed model failure cases or uncertainty estimation. Real-world deployment requires systems capable of recognizing and communicating their own limitations. Additionally, there is limited exploration of critical topics such as domain adaptation, cross-crop transfer learning, and privacy-preserving model learning. Furthermore, research on continuous model adaptation in dynamic field conditions remains limited, even as disease distributions evolve due to climatic and geographical changes.

## 7. Conclusions and future work

This work provides a comprehensive synthesis of DL approaches for plant disease diagnosis. It encompasses key topics including classification, detection, segmentation, explainability, data augmentation, real-world deployment, and architectural innovations. The reviewed studies collectively demonstrate how DL has revolutionized plant disease recognition, while simultaneously revealing persistent limitations and open research challenges.

The application of DL methods to plant disease detection has gained substantial attention owing to their ability to automatically extract discriminative features from complex visual data, eliminating the need for manual feature engineering. The reviewed literature indicates that convolutional neural network (CNN)-based architectures have substantially advanced the field, achieving high accuracy in both classification and localization across diverse plant diseases and image datasets. Beyond standard classification tasks, segmentation frameworks have enabled precise identification of diseased regions at the pixel level. Simultaneously, object detection models offer real-time localization capabilities that directly support modern precision agriculture systems.

Moreover, the growing emphasis on interpretability through visualization techniques—such as Grad-CAM, saliency maps, and heatmaps—enhances user trust and model transparency. Hybrid and attention-based architectures further improve feature extraction by enabling networks to selectively focus on spatial and channel-specific disease cues—an ability particularly valuable when symptoms are visually subtle or context-dependent. Despite these impressive advances, bridging the gap between laboratory-level accuracy and reliable real-world performance remains a persistent challenge. Most models continue to be evaluated on controlled datasets with uniform lighting and minimal background variability—such as PlantVillage—which fail to fully represent complex field conditions. Environmental factors—such as occlusions, soil background noise, and varying illumination—significantly affect model performance when applied to real-world imagery.

The cost of annotating data for segmentation and recognition tasks remains prohibitively high, particularly for small-scale farmers and research institutions with limited access to large annotated datasets. The reviewed studies emphasize that without diverse, high-quality, field-captured image data, DL models tend to overfit to region-specific features, thereby reducing their generalizability across different crop types, geographical regions, and agricultural practices. Several research opportunities and practical challenges are expected to shape future exploration of DL in plant disease diagnosis. First, there is an urgent need to develop and openly share comprehensive, well-annotated, and diverse field datasets. Such datasets should include images captured under diverse environmental conditions, across multiple regions, and at different growth stages to enable models to generalize effectively beyond controlled laboratory settings.

Furthermore, model compression techniques—such as pruning, quantization, and knowledge distillation—will be crucial for developing lightweight and efficient DL architectures suitable for deployment on mobile and embedded devices in low-resource agricultural environments. Optimizing energy efficiency and inference latency is particularly critical for real-time diagnostic systems operating in regions with unreliable or limited internet connectivity.

Another promising direction involves integrating diverse and complementary data sources. Combining RGB imagery with hyperspectral, thermal, LiDAR, and UAV-based data can provide complementary spectral and spatial information, enabling models to better distinguish between biotic and abiotic stress factors and to detect early-stage symptoms imperceptible to the human eye. Advances in self-supervised learning hold great potential to reduce dependence on large labeled datasets by enabling models to learn robust feature representations directly from unlabeled data. Similarly, federated learning frameworks facilitate collaborative model training across multiple farms and research institutions without the need to centralize raw data. This approach preserves data privacy while promoting knowledge sharing across distributed agricultural networks.

Ultimately, the importance of explainability and transparency in agricultural AI systems cannot be overstated. Developing unified architectures that seamlessly integrate classification, detection, segmentation, and explainability will yield trustworthy and actionable AI systems for farmers, agronomists, and policymakers. Beyond algorithmic enhancement, interdisciplinary collaboration with plant pathologists and agronomists is essential to ensure that DL models remain both biologically meaningful and practically applicable across diverse agricultural environments.

## Figures and Tables

**Table 1:** Open-source tools for deep learning.

Framework	Developer	Support	Interface	Usability
Theano	MILA	CPU, GPU	Python	Provides efficient symbolic expression handling, GPU acceleration, and support for parallel and distributed computing through an extensive mathematical function library.
Tensorflow	Google	CPU, GPU, Mobile	C, Python, Java	Offers flexibility with high-level APIs, cross-platform portability, and access to a wide range of pretrained models for deep learning research.
Pytorch	Meta AI (formerly Facebook)	CPU, GPU, FPGA	C, Python, Lua	Features dynamic neural network construction, easy debugging, strong community engagement, and rapidly expanding ecosystem.
Caffe	BAIR	CPU, GPU	Python, MATLAB	Enables fast execution with low memory consumption and benefits from a large, active open-source community.

**Table 2:** Summary of plant disease classification using visualization techniques.

Study	DL model	Accuracy (%)	Key findings
Brahimi et al. (2018)	CaffeNet CNN	99.76%	Utilized saliency maps to classify 13 distinct plant diseases.
Atila et al. (2021)	EfficientNet-B5/B4	99.91%–99.97%	Applied transfer learning with data augmentation using EfficientNet-B5 and B4 architectures.
Nagasubramanian et al. (2019)	3D DCNN	95.73%	Employed 3D deep convolutional neural networks with saliency maps for soybean stem and rot disease classification.
Gonzalez-Huitron et al. (2021)	Transfer learning (CNN-based)	N/A	Evaluated and compared four CNN models for tomato leaf disease detection.
Anim-Ayeko et al. (2023)	ResNet-9	99.25%	Applied data augmentation and hyperparameter tuning to improve ResNet-9 performance for plant disease classification.
Sun et al. (2019)	SVM with saliency maps	98.5%	Utilized saliency and morphological image features for plant disease classification.
Tembhurne et al. (2023)	MobileNet with saliency maps	95.94%	Utilized MobileNet architecture with hidden layers fine-tuned through saliency maps for plant disease classification.
Khan et al. (2020)	VGG-19, VGG-M + SVM	98.08%	Applied saliency-based image segmentation integrated with SVM classification for plant disease detection.
Wiesner-Hanks et al. (2019)	UAV-based CNN	99.79%	Applied heatmap-based CNNs for lesion classification in maize leaf diseases captured via UAV imagery.
Albattah et al. (2022)	CenterNet + DenseNet-77	N/A	Utilized the CenterNet framework integrated with DenseNet-77 features for plant disease categorization.
DeChant et al. (2017)	CNN Pipeline	96.7%	Developed a heatmap-based CNN pipeline for early-stage plant disease detection.
Nawaz et al. (2024)	CenterNet + ResNet-50	98.54%	Integrated spatial and channel attention mechanisms into the CenterNet–ResNet-50 architecture to enhance feature extraction.

**Table 3:** Summary of plant disease classification without visualization techniques.

Study	DL Model	Accuracy (%)	Key findings
Naik et al. (2022)	VGG-19	83.54%	Evaluated 12 CNN architectures for chili plant disease classification.
Dai et al. (2024)	DFN-PSAN	95.27%	Introduced a pyramidal attention mechanism within the DFN-PSAN model for fine-grained plant disease classification.
Dey et al. (2022)	VGG-19	91.8%	Demonstrated that simple CNN models can effectively classify nutrient-deficient leaves.
Faisal et al. (2023)	EfficientNetB3, ResNet-50, MobileNetV2	N/A	Utilized transfer learning with multiple DL architectures for citrus plant disease classification.
Alirezazadeh et al. (2023)	EfficientNetB0 + CBAM	86.89%	Integrated CBAM into EfficientNetB0 to enhance CNN performance in plant disease detection.
Amin et al. (2022)	EfficientNetB0, DenseNet121	98.56%	Performed feature extraction using EfficientNetB0 and DenseNet121 models on corn plant images for disease identification.
Oppenheim et al. (2019)	VGG	N/A	Classified images of infected potato leaves captured under natural light conditions using a VGG-based CNN.
Abbas et al. (2021)	DenseNet121 + C-GAN	N/A	Employed a combination of DenseNet121 and C-GAN to enhance classification accuracy.
Hassan et al. (2021)	InceptionV3, ResNetV2, EfficientNetB0	99.56%	Replaced standard convolution layers with depthwise separable convolution to optimize computational efficiency and accuracy.
Chen et al. (2021)	MobileNet-V2	99.67%	Applied an attention mechanism and transfer learning strategy using the MobileNet-V2 architecture for improved plant disease classification.
Ahad et al. (2023)	DenseNet121, MobileNetV2, ResNet152V	N/A	Applied ensemble learning combining DenseNet121, MobileNetV2, and ResNet152V for rice plant disease classification.
Pradhan et al. (2022)	DenseNet201	98.75%	Compared 10 CNN models for apple leaf disease classification.
Nagaraju and Chawla (2023)	NPNet-19	97.51%	Proposed a new deep CNN architecture, NPNet-19, for maize leaf disease classification.
Assad et al. (2023)	ResNet50 (AppleNet)	96.00%	Proposed AppleNet, a modified ResNet50-based CNN for apple disease classification.
Garg and Alam (2022)	Xception + LSTM	N/A	Developed a hybrid CNN and LSTM model for apple disease classification.
Bajait and Malarvizhi (2023)	SqueezeNet + SAE	96.31%	Employed hyperparameter optimization on a SqueezeNet–stacked autoencoder (SAE) framework for grape leaf disease classification.
Alsubai et al. (2023)	CNN-GRU	N/A	Applied the improved salp swarm optimization (ISSO) algorithm with a CNN-GRU hybrid network for grape leaf disease classification.

**Table 4:** Summary of one-stage plant disease detection models.

Study	Detection model	mAP (%)	Key findings
Qiang et al. (2023)	SSD	72.54% (single backbone) / 86.01% (dual backbone)	Achieved robust detection performance for citrus pest and disease recognition using a dual-backbone SSD architecture.
Guo et al. (2021)	Custom YOLO-like Detector, SSD	85.03%	Implemented multiresolution detection using ADAMW optimization and dropout regularization, improving detection generalization.
Saleem et al. (2022)	SSD	91.33%	Applied weight-optimized transfer learning, achieving improved mAP scores on the PlantVillage and NZDL benchmark datasets.
Saleem et al. (2020)	SSD	73.07%	Achieved the best mAP using the Adam optimizer among SSD, Faster R-CNN, and RFCN architectures.
Sun et al. (2020)	SSD	91.83%	Implemented multiscale feature fusion, improving SSD’s mAP from 71.8% to 91.83%.
Bao et al. (2023)	ASFFNet	80.8%	Proposed an adaptive spatial feature fusion network (ASFFNet) that outperformed SSD, YOLOv3, YOLOv4, and RetinaNet models in detection accuracy.

**Table 5:** Summary of two-stage plant disease detection models

Study	Model	mAP (%)	Key findings
Bari et al. (2021)	Faster R-CNN	99.17%, 98.85%, 98.09%	Achieved real-time detection of rice diseases—hispa, brown spot, and rice blast—using an enhanced RPN trained on both public and real-field datasets.
Gong and Zhang (2023)	Faster R-CNN	92.40%	Applied Faster R-CNN to apple leaf disease detection, outperforming YOLOv3 in complex leaf disease scenarios requiring precise lesion localization.
Alruwaili et al. (2022)	RTF-RCNN (Modified Faster R-CNN)	97.42%	Achieved real-time tomato leaf disease detection from both static images and video streams using a modified Faster R-CNN, outperforming AlexNet (96.32%) and conventional CNN models (92.21%).
Ozguven and Adem (2019)	Faster R-CNN	95.48%	Applied Faster R-CNN for sugar beet leaf spot disease detection, demonstating robustness under natural illumination and background variations.
Mu et al. (2022)	Faster RCNN + FPN	*>*95.00%	Employed a FPN-enhanced Faster R-CNN model for weed seedling detection, demonstrating improved capability to distinguish crops from weeds in complex field imagery.

**Table 6:** Description of commonly used leaf disease datasets.

Dataset	Number of images	Number of classes
**PlantVillage** 8	61,486	38
**Leaf image database** 9	4503	22
**LeafSnap** 10	30,866	185
**ImageCLEF** 11	11,572	3
**PlantDoc** (Singh et al., 2020)^12^	2598	13
**Plant disease symptom image database** (Garcia Arnal Barbedo et al., 2018)^13^	46,513	171
**Rice leaf disease dataset** (Shah et al., 2019)^14^	120	3
**Flavia** (Wu et al., 2007)^15^	1907	33
**DiaMOS** (Fenu and Malloci, (2021)^16^	3505	4
**Field plant dataset** (Moupojou et al., (2023)^17^	8629	27
**New plant disease dataset** 18	87,900	38
**Crop pest and disease detection dataset** (Mensah et al., 2023)^19^	102,976	22
**Plant pathology dataset** (Thapa et al., 2020)^20^	3645	4
**Citrus plant disease dataset** (Rauf et al., 2019)^21^	759	2
**BRACOL dataset** (Esgario et al., 2019)^22^	2147	5
**Apple leaf disease detection (ALDD) dataset** (Jiang et al., 2019)	2029	5
**Kashmiri apple plant disease dataset** (Sharma et al., 2022)^23^	419	4
**Groundnut leaf image dataset** (Aishwarya and Reddy, 2023)^24^	10,361	6
**Arabica coffee leaf dataset** (Jepkoech et al., (2021)^25^	58,555	5
**Tobacco plant disease dataset** (Lin et al., 2022)^26^	2721	12
**Robusta coffee leaf image dataset** (Lin et al., 2022)^27^	1560	2
**Black gram leaf disease dataset** (Talasila et al., 2022)^28^	1000	5
**Sunflower fruit and leaf dataset** (Sara et al., 2022)^29^	1668	4
**Bangladeshi crop disease dataset** 30	13,024	14
**Wheat disease database** (Long et al., (2023)^31^	19,172	5
**New Zealand fungal and plant disease collection** (Wilton, 2019)^32^	109,863	6
**Sugarcane leaf disease dataset** (Daphal and Koli, 2023)^33^	2569	5
**Sugarcane leaf disease dataset** (Huang and Chang, 2020)^34^	14,531	10

## Footnotes

1 PlantVillage (2019). PlantVillage dataset [online]. Website: https://data.mendeley.com/datasets/tywbtsjrjv/1 [accessed 01.10.2023]

2 Database of Leaf Images (2019). Database of Leaf Images [online]. Website: https://data.mendeley.com/datasets/hb74ynkjcn/4/ [accessed 01.10.2023]

3 LeafSnap (2014). LeafSnap dataset [online]. Website: http://leafsnap.com/dataset/ [accessed 01.10.2023]

4 ImageCLEF (2014). ImageCLEF dataset [online]. Website: https://www.imageclef.org/2012/plant [accessed 01.10.2023]

5 New Plant Diseases Dataset (2018). New Plant Diseases Dataset [online]. Website: https://www.kaggle.com/datasets/vipoooool/new-plant-diseases-dataset [accessed 01.10.2023]

6 Plant disease recognition dataset (2021), Plant disease recognition dataset [online]. Website: https://www.kaggle.com/datasets/rashikrahmanpritom/plant-disease-recognition-dataset [accessed 01.10.2023].

7 New Bangladeshi Crop Disease Dataset (2021). New Bangladeshi Crop Disease Dataset [online]. Website: https://www.kaggle.com/datasets/nafishamoin/new-bangladeshi-crop-disease [accessed 01.10.2023]

8 PlantVillage (2019). PlantVillage dataset [online]. Website: https://data.mendeley.com/datasets/tywbtsjrjv/1 [accessed 01.10.2023]

9 Database of Leaf Images (2019). Database of Leaf Images [online]. Website: https://data.mendeley.com/datasets/hb74ynkjcn/4/ [accessed 01.10.2023]

10 LeafSnap (2014). LeafSnap dataset [online]. Website: http://leafsnap.com/dataset/ [accessed 01.10.2023]

11 ImageCLEF (2014). ImageCLEF dataset [online]. Website: https://www.imageclef.org/2012/plant [accessed 01.10.2023]

12 PlantDoc (2020). PlantDoc dataset [online]. Website: https://github.com/pratikkayal/PlantDoc-Dataset [accessed 01.10.2023]

13 Image Database of Plant Disease Symptoms (2018). Image Database of Plant Disease Symptoms dataset [online]. Website: https://www.digipathos-rep.cnptia.embrapa.br/ [accessed 01.10.2023]

14 Rice Leaf Disease Dataset (2019). Rice Leaf Disease Dataset [online]. Website: https://archive.ics.uci.edu/dataset/486/rice+leaf+diseases [accessed 01.10.2023]

15 Flavia (2017). Flavia dataset [online]. Website: https://flavia.sourceforge.net/ [accessed 01.10.2023]

16 DiaMOS (2021). DiaMOS dataset [online]. Website: https://zenodo.org/records/5557313 [accessed 01.10.2023

17 Field Plant (2021). Field Plant dataset [online]. Website: https://ieeexplore.ieee.org/stamp/stamp.jsp?tp=&arnumber=100865163 [accessed 01.10.2023]

18 New Plant Diseases Dataset (2018). New Plant Diseases Dataset [online]. Website: https://www.kaggle.com/datasets/vipoooool/new-plant-diseases-dataset [accessed 01.10.2023]

19 Dataset for crop pest and disease detection (2023). Dataset for crop pest and disease detection dataset [online]. Website: https://data.mendeley.com/datasets/bwh3zbpkpv/1 [accessed 01.10.2023]

20 Plant Pathology Dataset (2020). Plant Pathology Dataset [online]. Website: https://www.kaggle.com/c/plant-pathology-2020-fgvc7/data [accessed 01.10.2023]

21 Citrus Plant Dataset (2019). Citrus Plant Dataset [online]. Website: https://data.mendeley.com/datasets/3f83gxmv57/2 [accessed 01.10.2023]

22 BRACOL Dataset (2019). BRACOL Dataset [online]. Website: https://data.mendeley.com/datasets/yy2k5y8mxg/1 [accessed 01.10.2023]

23 Kashimiri Apple Plant Disease Dataset (2022). Kashimiri Apple Plant Disease Dataset [online]. Website: https://www.kaggle.com/datasets/hsmcaju/d-kap [accessed 01.10.2023]

24 Dataset of groundnut plant leaf images (2023). Dataset of groundnut plant leaf images [online]. Website: https://data.mendeley.com/datasets/22p2vcbxfk/3 [accessed 01.10.2023]

25 Arabica coffee leaf dataset (2021). Arabica coffee leaf dataset [online]. Website: https://data.mendeley.com/datasets/tgv3zb82nd/1 [accessed 01.10.2023]

26 Tobacco plant disease dataset (2022). Tobacco plant disease dataset [online]. Website: https://github.com/honglin1226/Tobacco-Plant-Disease-Dataset [accessed 01.10.2023]

27 Robusta Coffee Leaf Images Dataset (2022). Robusta Coffee Leaf Images Dataset [online]. Website: https://data.mendeley.com/datasets/c5yvn32dzg/2

28 Black gram Plant Leaf Disease Dataset (2022). Black gram Plant Leaf Disease Dataset [online]. Website: https://data.mendeley.com/datasets/zfcv9fmrgv/3 [accessed 01.10.2023]

29 Sun flower fruits and leaves dataset (2022). Sun flower fruits and leaves dataset [online]. Website: https://data.mendeley.com/datasets/b83hmrzth8/1 [accessed 01.10.2023]

30 New Bangladeshi Crop Disease Dataset (2021). New Bangladeshi Crop Disease Dataset [online]. Website: https://www.kaggle.com/datasets/nafishamoin/new-bangladeshi-crop-disease [accessed 01.10.2023]

31 Wheat disease database (2023). Wheat disease database [online]. Website: https://zenodo.org/records/7573133 [accessed 01.10.2023]

32 New Zealand Fungal and Plant Disease Collection (2019). New Zealand Fungal and Plant Disease Collection dataset [online]. Website: https://www.gbif.org/dataset/ee27b1b0-3b55-11dc-8c18-b8a03c50a862 [accessed 01.10.2023]

33 Sugarcane Leaf Disease Dataset (2023). Sugarcane Leaf Disease Dataset [online]. Website: https://data.mendeley.com/datasets/9424skmnrk/1 [accessed 01.10.2023]

34 Sugarcane Leaf Disease Dataset (2020). Sugarcane Leaf Disease Dataset [online]. Website: https://data.mendeley.com/datasets/ngdgg79rzb/1 [accessed 01.10.2023]

## References

[b1-tjb-49-05-459] Abbas A Jain S Gour M Vankudothu S 2021 Tomato plant disease detection using transfer learning with c-gan synthetic images Computers and Electronics in Agriculture 187 106279 10.1016/j.compag.2021.106279

[b2-tjb-49-05-459] Abd Algani YM Marquez Caro OJ Robladillo Bravo LM Kaur C Al Ansari MS 2023 Leaf disease identification and classification using optimized deep learning Measurement: Sensors 25 100643 10.1016/j.measen.2022.100643

[b3-tjb-49-05-459] Adnan F Awan MJ Mahmoud A Nobanee H Yasin A 2023 Efficientnetb3-adaptive augmented deep learning (aadl) for multi-class plant disease classification IEEE Access 11 85426 85440 10.1109/ACCESS.2023.3303131

[b4-tjb-49-05-459] Ahad MT Li Y Song B Bhuiyan T 2023 Comparison of cnn-based deep learning architectures for rice diseases classification Artificial Intelligence in Agriculture 9 22 35 10.1016/j.aiia.2023.07.001

[b5-tjb-49-05-459] Ahmad M Abdullah M Moon H Han D 2021 Plant disease detection in imbalanced datasets using efficient convolutional neural networks with stepwise transfer learning IEEE Access 9 140565 140580 10.1109/ACCESS.2021.3119655

[b6-tjb-49-05-459] Aishwarya M Reddy AP 2023 Dataset of groundnut plant leaf images for classification and detection Data in Brief 48 109185 10.1016/j.dib.2023.109185 37383808 PMC10293989

[b7-tjb-49-05-459] Albattah W Nawaz M Javed A Masood M Albahli S 2022 A novel deep learning method for detection and classification of plant diseases Complex & Intelligent Systems 8 507 524 10.1007/s40747-021-00536-1

[b8-tjb-49-05-459] Ali U Ismail MA Habeeb RAA Shah SRA 2024 Performance evaluation of yolo models in plant disease detection Journal of Informatics and Web Engineering 3 199 211

[b9-tjb-49-05-459] Alirezazadeh P Schirrmann M Stolzenburg F 2023 Improving deep learning-based plant disease classification with attention mechanism Gesunde Pflanzen 75 49 59 10.1007/s10343-022-00796-y

[b10-tjb-49-05-459] Alruwaili M Siddiqi MH Khan A Azad M Khan A 2022 Rtf-rcnn: An architecture for real-time tomato plant leaf diseases detection in video streaming using faster-rcnn Bioengineering 9 10.3390/bioengineering9100565 PMC9598809 36290533

[b11-tjb-49-05-459] Alsubai S Dutta AK Alkhayyat AH Jaber MM Abbas AH 2023 Hybrid deep learning with improved salp swarm optimization based multi-class grape disease classification model Computers and Electrical Engineering 108 108733 10.1016/j.compeleceng.2023.108733

[b12-tjb-49-05-459] Altalak M Uddin MA Alajmi A Rizg A 2022 A hybrid approach for the detection and classification of tomato leaf diseases Applied Sciences 12 10.3390/app12168182

[b13-tjb-49-05-459] Amin H Darwish A Hassanien AE Soliman M 2022 End-to-end deep learning model for corn leaf disease classification IEEE Access 10 31103 31115

[b14-tjb-49-05-459] Andrew J Eunice J Popescu DE Chowdary MK Hemanth J 2022 Deep learning-based leaf disease detection in crops using images for agricultural applications Agronomy 12 10.3390/agronomy12102395

[b15-tjb-49-05-459] Anim-Ayeko AO Schillaci C Lipani A 2023 Automatic blight disease detection in potato (solanum tuberosum l. and tomato (solanum lycopersicum, l. 1753) plants using deep learning Smart Agricultural Technology 4 100178 10.1016/j.atech.2023.100178

[b16-tjb-49-05-459] Arnal Barbedo JG 2019 Plant disease identification from individual lesions and spots using deep learning Biosystems Engineering 180 96 107 10.1016/j.biosystemseng.2019.02.002

[b17-tjb-49-05-459] Arshaghi A Ashourian M Ghabeli L 2023 Potato diseases detection and classification using deep learning methods Multimedia Tools and Applications 82 5725 5742 10.1007/s11042-022-13390-1

[b18-tjb-49-05-459] Assad A Bhat MR Bhat Z Ahanger AN Kundroo M 2023 Apple diseases: detection and classification using transfer learning Quality Assurance and Safety of Crops & Foods 15 27 37 10.15586/qas.v15iSP1.1167

[b19-tjb-49-05-459] Atila Ü Ucar M Akyol K Ucar E 2021 Plant leaf disease classification using efficientnet deep learning model Ecological Informatics 61 101182 10.1016/j.ecoinf.2020.101182

[b20-tjb-49-05-459] Bajait V Malarvizhi N 2023 Automated grape leaf nutrition deficiency disease detection and classification equilibrium optimizer with deep transfer learning model Network: Computation in Neural Systems 0 1 18 10.1080/0954898X.2023.2275722 37933604

[b21-tjb-49-05-459] Bao W Liu W Yang X Hu G Zhang D 2023 Adaptively spatial feature fusion network: an improved uav detection method for wheat scab Precision Agriculture 24 1154 1180 10.1007/s11119-023-10004-0

[b22-tjb-49-05-459] Barbedo JGA 2018 Impact of dataset size and variety on the effectiveness of deep learning and transfer learning for plant disease classification Computers and Electronics in Agriculture 153 46 53 10.1016/j.compag.2018.08.013

[b23-tjb-49-05-459] Bari BS Islam MN Rashid M Hasan MJ Razman MAM 2021 A real-time approach of diagnosing rice leaf disease using deep learning-based faster r-cnn framework PeerJ Computer Science 7e432 10.7717/peerj-cs.432 PMC8049121 33954231

[b24-tjb-49-05-459] Bhujade VG Sambhe V Banerjee B 2024 Digital image noise removal towards soybean and cotton plant disease using image processing filters Expert Systems with Applications 246 123031 10.1016/j.eswa.2023.123031

[b25-tjb-49-05-459] Bi L Hu G 2020 Improving image-based plant disease classification with generative adversarial network under limited training set Frontiers in Plant Science 11 583438 10.3389/fpls.2020.583438 33343595 PMC7746658

[b26-tjb-49-05-459] Brahimi M Arsenovic M Laraba S Sladojevic S Boukhalfa K 2018 Deep Learning for Plant Diseases: Detection and Saliency Map Visualisation Springer International Publishing Cham 93 117 10.1007/978-3-319-90403-0_6

[b27-tjb-49-05-459] Canziani A Paszke A Culurciello E 2017 An analysis of deep neural network models for practical applications 10.48550/arXiv.1605.07678

[b28-tjb-49-05-459] Cao Y Yuan P Xu H Martinez-Ortega JF Feng J 2022 Detecting asymptomatic infections of rice bacterial leaf blight using hyperspectral imaging and 3-dimensional convolutional neural network with spectral dilated convolution Frontiers in Plant Science 13 10.3389/fpls.2022.963170 PMC9328758 35909723

[b29-tjb-49-05-459] Cetinkaya S Tandirovic Gursel A 2025 Pep-vggnet: A novel transfer learning method for pepper leaf disease diagnosis Applied Sciences 15 10.3390/app15158690

[b30-tjb-49-05-459] Chaudhari P Patil RV Mahalle PN 2023 Early phase identification and detection for plant poor growth in rural areas: A survey of the state of the art Choudrie J Mahalle PN Perumal T IOT with Smart Systems Springer Nature Singapore Singapore 29 42

[b31-tjb-49-05-459] Chen J Chen J Zhang D Sun Y Nanehkaran Y 2020 Using deep transfer learning for image-based plant disease identification Computers and Electronics in Agriculture 173 105393 10.1016/j.compag.2020.105393

[b32-tjb-49-05-459] Chen J Zhang D Zeb A Nanehkaran YA 2021 Identification of rice plant diseases using lightweight attention networks Expert Systems with Applications 169 114514 10.1016/j.eswa.2020.114514

[b33-tjb-49-05-459] Chowdhury MEH Rahman T Khandakar A Ayari MA Khan AU 2021 Automatic and reliable leaf disease detection using deep learning techniques AgriEngineering 3 294 312 10.3390/agriengineering3020020

[b34-tjb-49-05-459] Chug A Bhatia A Singh AP Singh D 2023 A novel framework for image-based plant disease detection using hybrid deep learning approach Soft Computing 27 13613 13638 10.1007/s00500-022-07177-7

[b35-tjb-49-05-459] Corlouer E Sauvage C Leveugle M Nesi N Laperche A 2024 Envirotyping within a multi-environment trial allowed identifying genetic determinants of winter oilseed rape yield stability Theoretical and Applied Genetics 137 164 10.1007/s00122-024-04664-3 38898332 PMC11186914

[b36-tjb-49-05-459] Coulibaly S Kamsu-Foguem B Kamissoko D Traore D 2019 Deep neural networks with transfer learning in millet crop images Computers in Industry 108 115 120 10.1016/j.compind.2019.02.003

[b37-tjb-49-05-459] Dai G Tian Z Fan J Sunil C Dewi C 2024 Dfn-psan: Multi-level deep information feature fusion extraction network for interpretable plant disease classification Computers and Electronics in Agriculture 216 108481 10.1016/j.compag.2023.108481

[b38-tjb-49-05-459] Daphal SD Koli SM 2023 Enhancing sugarcane disease classification with ensemble deep learning: A comparative study with transfer learning techniques Heliyon 9 10.17632/9424skmnrk.1 PMC10382639 37520940

[b39-tjb-49-05-459] DeChant C Wiesner-Hanks T Chen S Stewart EL Yosinski J 2017 Automated identification of northern leaf blight-infected maize plants from field imagery using deep learning Phytopathology 107 1426 1432 10.1094/PHYTO-11-16-0417-R 28653579

[b40-tjb-49-05-459] Deng J Dong W Socher R Li LJ Li K 2009 Imagenet: A large-scale hierarchical image database 2009 IEEE Conference on Computer Vision and Pattern Recognition 248 255 10.1109/CVPR.2009.5206848

[b41-tjb-49-05-459] Dey B Masum Ul Haque M Khatun R Ahmed R 2022 Comparative performance of four cnn-based deep learning variants in detecting hispa pest, two fungal diseases, and npk deficiency symptoms of rice (oryza sativa) Computers and Electronics in Agriculture 202 107340 10.1016/j.compag.2022.107340

[b42-tjb-49-05-459] Dinc B Kaya Y 2024 Hbdfa: An intelligent nature-inspired computing with high-dimensional data analytics Multimedia Tools and Applications 83 11573 11592 10.1007/s11042-023-16039-9

[b43-tjb-49-05-459] Dong X Wang Q Huang Q Ge Q Zhao K 2023 Pddd-pretrain: A series of commonly used pre-trained models support image-based plant disease diagnosis Plant Phenomics 5 0054 10.34133/plantphenomics.0054 37213546 PMC10194370

[b44-tjb-49-05-459] Esgario JGM Krohling RA Ventura JA 2019 Deep learning for classification and severity estimation of coffee leaf biotic stress

[b45-tjb-49-05-459] Faisal S Javed K Sial S Alasiry A Marzougui M 2023 Deep transfer learning based detection and classification of citrus plant diseases Computers, Materials and Continua 76 10.32604/cmc.2023.039781

[b46-tjb-49-05-459] Fan X Luo P Mu Y Zhou R Tjahjadi T 2022 Leaf image based plant disease identification using transfer learning and feature fusion Computers and Electronics in Agriculture 196 106892 10.1016/j.compag.2022.106892

[b47-tjb-49-05-459] FAO 2021 The impact of disasters and crises on agriculture and food security Food and Agriculture Organization 10.4060/cb3673en

[b48-tjb-49-05-459] Feng L Wu B He Y Zhang C 2021 Hyperspectral imaging combined with deep transfer learning for rice disease detection Frontiers in Plant Science 12 10.3389/fpls.2021.693521 PMC8511421 34659278

[b49-tjb-49-05-459] Fenu G Malloci FM 2021 Diamos plant: A dataset for diagnosis and monitoring plant disease Agronomy 11 10.3390/agronomy11112107

[b50-tjb-49-05-459] Ferentinos KP 2018 Deep learning models for plant disease detection and diagnosis Computers and Electronics in Agriculture 145 311 318 10.1016/j.compag.2018.01.009

[b51-tjb-49-05-459] Fraiwan M Faouri E Khasawneh N 2022 Classification of corn diseases from leaf images using deep transfer learning Plants 11 10.3390/plants11202668 PMC9609100 36297692

[b52-tjb-49-05-459] Gao W Xiao Z Bao T 2023 Detection and identification of potato-typical diseases based on multidimensional fusion atrous-cnn and hyperspectral data Applied Sciences 13 10.3390/app13085023

[b53-tjb-49-05-459] Garcia Arnal Barbedo J Vieira Koenigkan L Almeida Halfeld-Vieira B Veras Costa R Lima Nechet K 2018 Annotated plant pathology databases for image-based detection and recognition of diseases IEEE Latin America Transactions 16 1749 1757 10.1109/TLA.2018.8444395

[b54-tjb-49-05-459] Garg D Alam M 2022 Integration of convolutional neural networks and recurrent neural networks for foliar disease classification in apple trees International Journal of Advanced Computer Science and Applications 13 10.14569/IJACSA.2022.0130442

[b55-tjb-49-05-459] Gong X Zhang S 2023 An analysis of plant diseases identification based on deep learning methods The Plant Pathology Journal 39 319 10.5423/PPJ.OA.02.2023.0034 37550979 PMC10412967

[b56-tjb-49-05-459] Gonzalez-Huitron V Leon-Borges JA Rodriguez-Mata A Amabilis-Sosa LE Ramirez-Pereda B 2021 Disease detection in tomato leaves via cnn with lightweight architectures implemented in raspberry pi 4 Computers and Electronics in Agriculture 181 105951 10.1016/j.compag.2020.105951

[b57-tjb-49-05-459] Goodfellow I Bengio Y Courville A Bengio Y 2016 Deep learning. volume 1 MIT press Cambridge

[b58-tjb-49-05-459] Guan H Fu C Zhang G Li K Wang P 2023 A lightweight model for efficient identification of plant diseases and pests based on deep learning Frontiers in Plant Science 14 1227011 10.3389/fpls.2023.1227011 37521914 PMC10382237

[b59-tjb-49-05-459] Guo D Liu J Wang X 2021 On development of multi-resolution detector for tomato disease diagnosis Journal of Intelligent & Fuzzy Systems 41 6461 6471

[b60-tjb-49-05-459] Gursoy E Kaya Y 2024 Multi-source deep feature fusion for medical image analysis Multidimensional Systems and Signal Processing 36 4 10.1007/s11045-024-00897-z

[b61-tjb-49-05-459] Hamroun M Lajmi S Jallouli M 2025 Avr (advancing video retrieval): A new framework guided by multi-level fusion of visual and semantic features for deep learning-based concept detection Multimedia Tools and Applications 84 2715 2777 10.1007/s11042-024-20112-2

[b62-tjb-49-05-459] Hasan MJ Mahbub S Alom MS Abu Nasim M 2019 Rice disease identification and classification by integrating support vector machine with deep convolutional neural network 2019 1st International Conference on Advances in Science, Engineering and Robotics Technology (ICASERT) 1 6 10.1109/ICASERT.2019.8934568

[b63-tjb-49-05-459] Hassan SM Maji AK Jasinski M Leonowicz Z Jasinska E 2021 Identification of plant-leaf diseases using cnn and transfer-learning approach Electronics 10 10.3390/electronics10121388

[b64-tjb-49-05-459] Hayit T Endes A Hayit F 2023 The severity level classification of fusarium wilt of chickpea by pre-trained deep learning models Journal of Plant Pathology 10.1007/s42161-023-01520-z

[b65-tjb-49-05-459] Hinton GE Osindero S Teh YW 2006 A fast learning algorithm for deep belief nets Neural Computation 18 1527 1554 10.1162/neco.2006.18.7.1527 16764513

[b66-tjb-49-05-459] Huang ML Chang YH 2020 Dataset of tomato leaves Mendeley Data 1 2020 DOI: 10.17632/zfv4jj7855.1

[b67-tjb-49-05-459] Isinkaye FO Olusanya MO Singh PK 2024 Deep learning and content-based filtering techniques for improving plant disease identification and treatment recommendations: A comprehensive review Heliyon 10 9 10.1016/j.heliyon.2024.e29583 PMC11088271 38737274

[b68-tjb-49-05-459] Islam M Dinh A Wahid K Bhowmik P 2017 Detection of potato diseases using image segmentation and multiclass support vector machine 2017 IEEE 30th Canadian Conference on Electrical and Computer Engineering (CCECE) 1 4 10.1109/CCECE.2017.7946594

[b69-tjb-49-05-459] Jepkoech J Mugo DM Kenduiywo BK Too EC 2021 Arabica coffee leaf images dataset for coffee leaf disease detection and classification Data in Brief 36 107142 doi: 10.1016/j.dib.2021.107142 34095388 PMC8165403

[b70-tjb-49-05-459] Jia Y Shelhamer E Donahue J Karayev S Long J 2014 Caffe: Convolutional architecture for fast feature embedding Proceedings of the 22nd ACM International Conference on Multimedia, Association for Computing Machinery New York, NY, USA 675 678 10.1145/2647868.2654889

[b71-tjb-49-05-459] Jiang P Chen Y Liu B He D Liang C 2019 Real-time detection of apple leaf diseases using deep learning approach based on improved convolutional neural networks IEEE Access 7 59069 59080 10.1109/ACCESS.2019.2914929

[b72-tjb-49-05-459] Kamilaris A Prenafeta-Boldú FX 2018 Deep learning in agriculture: A survey Computers and Electronics in Agriculture 147 70 90 10.1016/j.compag.2018.02.016

[b73-tjb-49-05-459] Karim MJ Goni MOF Nahiduzzaman M Ahsan M Haider J 2024 Enhancing agriculture through real-time grape leaf disease classification via an edge device with a lightweight cnn architecture and grad-cam Scientific Reports 14 16022 10.1038/s41598-024-66989-9 38992069 PMC11239930

[b74-tjb-49-05-459] Kaur P Harnal S Gautam V Singh MP Singh SP 2024 Performance analysis of segmentation models to detect leaf diseases in tomato plant Multimedia Tools and Applications 83 16019 16043 10.1007/s11042-023-16238-4

[b75-tjb-49-05-459] Kaya Y Akat E Yıldırım S 2025 Fusion-brain-net: A novel deep fusion model for brain tumor classification Brain and Behavior 15e70520 10.1002/brb3.70520 PMC12060224 40341828

[b76-tjb-49-05-459] Kaya Y Gursoy E 2023 A novel multi-head cnn design to identify plant diseases using the fusion of rgb images Ecological Informatics 75 10 1998 10.1016/j.ecoinf.2023.101998

[b77-tjb-49-05-459] Keller JA Shea K 2021 Warming and shifting phenology accelerate an invasive plant life cycle Ecology 102e03219 10.1002/ecy.3219 PMC7816242 33048356

[b78-tjb-49-05-459] Khan A Rauf Z Sohail A Khan AR Asif H 2023a A survey of the vision transformers and their cnn-transformer based variants Artificial Intelligence Review 56 2917 2970 10.1007/s10462-023-10595-0

[b79-tjb-49-05-459] Khan AT Jensen SM Khan AR Li S 2023b Plant disease detection model for edge computing devices Frontiers in Plant Science 14 1308528 doi: 10.3389/fpls.2023.1308528 38143571 PMC10748432

[b80-tjb-49-05-459] Khan MA Akram T Sharif M Javed K Raza M 2020 An automated system for cucumber leaf diseased spot detection and classification using improved saliency method and deep features selection Multimedia Tools and Applications 79 18627 18656 10.1007/s11042-020-08726-8

[b81-tjb-49-05-459] Khare O Mane S Kulkarni H Barve N 2024 Leafnst: an improved data augmentation method for classification of plant disease using object-based neural style transfer Discover Artificial Intelligence 4 50 10.1007/s44163-024-00150-3

[b82-tjb-49-05-459] Krizhevsky A Sutskever I Hinton G 2012 Imagenet classification with deep convolutional neural networks Pereira F Burges C Bottou L Weinberger K Advances in Neural Information Processing Systems Curran Associates, Inc 1 9

[b83-tjb-49-05-459] Kumar MS Ganesh D Turukmane AV Batta U Sayyadliyakat KK 2022 Deep convolution neural network based solution for detecting plant diseases Journal of Pharmaceutical Negative Results 13 464 471 10.47750/pnr.2022.13.S01.57

[b84-tjb-49-05-459] Lecun Y Bottou L Bengio Y Haffner P 1998 Gradient-based learning applied to document recognition Proceedings of the IEEE 86 2278 2324 10.1109/5.726791

[b85-tjb-49-05-459] Lee S Yun CM 2023 A deep learning model for predicting risks of crop pests and diseases from sequential environmental data Plant Methods 19 145 10.1186/s13007-023-01122-x 38093269 PMC10720067

[b86-tjb-49-05-459] Lin H Tse R Tang SK Qiang Z Ou J 2022 Tobacco plant disease dataset Fourteenth International Conference on Digital Image Processing (ICDIP 2022) 123423Q 10.1117/12.2644288

[b87-tjb-49-05-459] Long M Hartley M Morris RJ Brown JK 2023 Classification of wheat diseases using deep learning networks with field and glasshouse images Plant Pathology 72 536 547 10.1111/ppa.13684 38516179 PMC10953319

[b88-tjb-49-05-459] Lu Y Chen D Olaniyi E Huang Y 2022 Generative adversarial networks (gans) for image augmentation in agriculture: A systematic review Computers and Electronics in Agriculture 200 107208 10.1016/j.compag.2022.107208

[b89-tjb-49-05-459] Mathew MP Mahesh TY 2022 Leaf-based disease detection in bell pepper plant using yolo v5 Signal, Image and Video Processing 16 841 847 10.1007/s11760-021-02024-y

[b90-tjb-49-05-459] Mensah PK Akoto-Adjepong V Adu K Ayidzoe MA Bediako EA 2023 Ccmt: Dataset for crop pest and disease detection Data in Brief 49 109306 10.1016/j.dib.2023.109306 37360671 PMC10285554

[b91-tjb-49-05-459] Min B Kim T Shin D Shin D 2023 Data augmentation method for plant leaf disease recognition Applied Sciences 13 3 10.3390/app13031465

[b92-tjb-49-05-459] Mohameth F Bingcai C Sada KA 2020 Plant disease detection with deep learning and feature extraction using plant village Journal of Computer and Communications 8 10 22 10.4236/jcc.2020.86002

[b93-tjb-49-05-459] Mohanty SP Hughes DP Salathe M 2016 Using deep learning for image-based plant disease detection Frontiers in Plant Science 7 215232 10.3389/fpls.2016.01419 PMC5032846 27713752

[b94-tjb-49-05-459] Moupojou E Tagne A Retraint F Tadonkemwa A Wilfried D 2023 Fieldplant: A dataset of field plant images for plant disease detection and classification with deep learning IEEE Access 11 35398 35410 10.1109/ACCESS.2023.3263042

[b95-tjb-49-05-459] Mu Y Feng R Ni R Li J Luo T 2022 A faster r-cnn-based model for the identification of weed seedling Agronomy 12 10.3390/agronomy12112867

[b96-tjb-49-05-459] Muhammad A Salman Z Lee K Han D 2023 Harnessing the power of diffusion models for plant disease image augmentation Frontiers in Plant Science 14 1280496 10.3389/fpls.2023.1280496 38023884 PMC10669158

[b97-tjb-49-05-459] Mzoughi O Yahiaoui I 2023 Deep learning-based segmentation for disease identification Ecological Informatics 75 10 2000 10.1016/j.ecoinf.2023.102000

[b98-tjb-49-05-459] Nader A Khafagy MH Hussien SA 2022 Grape leaves diseases classification using ensemble learning and transfer learning International Journal of Advanced Computer Science and Applications 13 563 571 10.14569/IJACSA.2022.0130767

[b99-tjb-49-05-459] Nagaraju M Chawla P 2023 Maize crop disease detection using npnet-19 convolutional neural network Neural Computing and Applications 35 3075 3099 10.1007/s00521-022-07722-3

[b100-tjb-49-05-459] Nagasubramanian K Jones S Singh AK Sarkar S Singh A 2019 Plant disease identification using explainable 3d deep learning on hyperspectral images Plant Methods 15 98 10.1186/s13007-019-0479-8 31452674 PMC6702735

[b101-tjb-49-05-459] Naik BN Malmathanraj R Palanisamy P 2022 Detection and classification of chilli leaf disease using a squeeze-andexcitation-based cnn model Ecological Informatics 69 101663 10.1016/j.ecoinf.2022.101663

[b102-tjb-49-05-459] Nawaz M Nazir T Javed A Tawfik Amin S Jeribi F 2024 Coffeenet: A deep learning approach for coffee plant leaves diseases recognition Expert Systems with Applications 237 121481 10.1016/j.eswa.2023.121481

[b103-tjb-49-05-459] Neupane K Baysal-Gurel F 2021 Automatic identification and monitoring of plant diseases using unmanned aerial vehicles: A review Remote Sensing 13 10.3390/rs13193841

[b104-tjb-49-05-459] Ngugi HN Akinyelu AA Ezugwu AE 2024 Machine learning and deep learning for crop disease diagnosis: Performance analysis and review Agronomy 14 10.3390/agronomy14123001

[b105-tjb-49-05-459] Nguyen C Sagan V Maimaitiyiming M Maimaitijiang M Bhadra S 2021 Early detection of plant viral disease using hyperspectral imaging and deep learning Sensors 21 10.3390/s21030742 PMC7866105 33499335

[b106-tjb-49-05-459] Ogrekci S Unal Y Dudak MN 2023 A comparative study of vision transformers and convolutional neural networks: sugarcane leaf diseases identification European Food Research and Technology 249 1833 1843 10.1007/s00217-023-04258-1

[b107-tjb-49-05-459] Ojo MO Zahid A 2023 Improving deep learning classifiers performance via preprocessing and class imbalance approaches in a plant disease detection pipeline Agronomy 13 10.3390/agronomy13030887

[b108-tjb-49-05-459] Oppenheim D Shani G Erlich O Tsror L 2019 Using deep learning for image-based potato tuber disease detection Phytopathology 109 1083 1087 10.1094/PHYTO-08-18-0288-R 30543489

[b109-tjb-49-05-459] Ozguven MM Adem K 2019 Automatic detection and classification of leaf spot disease in sugar beet using deep learning algorithms Physica A Statistical Mechanics and its Applications 535 122537 10.1016/j.physa.2019.122537

[b110-tjb-49-05-459] Pan SJ Yang Q 2010 A survey on transfer learning IEEE Transactions on Knowledge and Data Engineering 22 1345 1359 10.1109/TKDE.2009.191

[b111-tjb-49-05-459] Park K ki Hong Y hwan Kim G Lee J 2018 Classification of apple leaf conditions in hyper-spectral images for diagnosis of marssonina blotch using mrmr and deep neural network Computers and Electronics in Agriculture 148 179 187 10.1016/j.compag.2018.02.025

[b112-tjb-49-05-459] Parraga-Alava J Cusme K Loor A Santander E 2019 Rocole: A robusta coffee leaf images dataset for evaluation of machine learning based methods in plant diseases recognition Data in Brief 25 104414 10.1016/j.dib.2019.104414 31516934 PMC6727496

[b113-tjb-49-05-459] Polder G Blok PM De Villiers HA Van der Wolf JM Kamp J 2019 Potato virus y detection in seed potatoes using deep learning on hyperspectral images Frontiers in Plant Science 10 209 10.3389/fpls.2019.00209 30881366 PMC6405642

[b114-tjb-49-05-459] Pradhan P Kumar B Mohan S 2022 Comparison of various deep convolutional neural network models to discriminate apple leaf diseases using transfer learning Journal of Plant Diseases and Protection 129 1461 1473 10.1007/s41348-022-00660-1

[b115-tjb-49-05-459] Qiang J Liu W Li X Guan P Du Y 2023 Detection of citrus pests in double backbone network based on single shot multibox detector Computers and Electronics in Agriculture 212 108158 10.1016/j.compag.2023.108158

[b116-tjb-49-05-459] Ramesh S Hebbar R Niveditha M Pooja R Prasad B 2018 Plant disease detection using machine learning 2018 International Conference on Design Innovations for 3Cs Compute Communicate Control (ICDI3C) 41 45 10.1109/ICDI3C.2018.00017

[b117-tjb-49-05-459] Rancon F Bombrun L Keresztes B Germain C 2019 Comparison of sift encoded and deep learning features for the classification and detection of esca disease in bordeaux vineyards Remote Sensing 11 10.3390/rs11010001

[b118-tjb-49-05-459] Rauf HT Saleem BA Lali MIU Khan MA Sharif M 2019 A citrus fruits and leaves dataset for detection and classification of citrus diseases through machine learning Data Brief 26 104340 10.1016/j.dib.2019.104340 31516936 PMC6731382

[b119-tjb-49-05-459] Saleem MH Khanchi S Potgieter J Arif KM 2020 Image-based plant disease identification by deep learning metaarchitectures Plants 9 10.3390/plants9111451 PMC7692455 33121188

[b120-tjb-49-05-459] Saleem MH Potgieter J Arif KM 2022 A weight optimization-based transfer learning approach for plant disease detection of new zealand vegetables Frontiers in Plant Science 13 1008079 10.3389/fpls.2022.1008079 36388538 PMC9641257

[b121-tjb-49-05-459] Sara U Rajbongshi A Shakil R Akter B Sazzad S 2022 An extensive sunflower dataset representation for successful identification and classification of sunflower diseases Data in Brief 42 108043 10.1016/j.dib.2022.108043 35392617 PMC8980537

[b122-tjb-49-05-459] Sarkar C Gupta D Gupta U Hazarika BB 2023 Leaf disease detection using machine learning and deep learning: Review and challenges Applied Soft Computing 145 110534 10.1016/j.asoc.2023.110534

[b123-tjb-49-05-459] Sethy PK Barpanda NK Rath AK Behera SK 2020 Deep feature based rice leaf disease identification using support vector machine Computers and Electronics in Agriculture 175 105527 10.1016/j.compag.2020.105527

[b124-tjb-49-05-459] Shafik W Tufail A Namoun A De Silva LC Apong RAAHM 2023 A systematic literature review on plant disease detection: Motivations, classification techniques, datasets, challenges, and future trends IEEE Access 11 59174 59203 10.1109/ACCESS.2023.3284760

[b125-tjb-49-05-459] Shah J Prajapati H Dabhi V 2019 Rice Leaf Diseases UCI Machine Learning Repository 10.24432/C5R013

[b126-tjb-49-05-459] Shao F Wang X Meng F Zhu J Wang D 2019 Improved faster r-cnn traffic sign detection based on a second region of interest and highly possible regions proposal network Sensors 19 10.3390/s19102288 PMC6567367 31108980

[b127-tjb-49-05-459] Sharma H Padha D Bashir N 2022 D-kap: A deep learning-based kashmiri apple plant disease prediction framework 2022 Seventh International Conference on Parallel, Distributed and Grid Computing (PDGC). 576 581 10.1109/PDGC56933.2022.10053334

[b128-tjb-49-05-459] Shoaib M Shah B Ei-Sappagh S Ali A Ullah A 2023 An advanced deep learning models-based plant disease detection: A review of recent research Frontiers in Plant Science 14 1158933 10.3389/fpls.2023.1158933 37025141 PMC10070872

[b129-tjb-49-05-459] Simhadri CG Kondaveeti HK 2023 Automatic recognition of rice leaf diseases using transfer learning Agronomy 13 10.3390/agronomy13040961

[b130-tjb-49-05-459] Simonyan K Zisserman A 2015 Very deep convolutional networks for large-scale image recognition 10.48550/arXiv.1409.1556

[b131-tjb-49-05-459] Singh AK Ganapathysubramanian B Sarkar S Singh A 2018 Deep learning for plant stress phenotyping: trends and future perspectives Trends in Plant Science 23 883 898 10.1016/j.tplants.2018.07.004 30104148

[b132-tjb-49-05-459] Singh D Jain N Jain P Kayal P Kumawat S 2020 Plantdoc: A dataset for visual plant disease detection Proceedings of the 7th ACM IKDD CoDS and 25th COMAD, Association for Computing Machinery New York, NY, USA 249 253 10.1145/3371158.3371196

[b133-tjb-49-05-459] Sladojevic S Arsenovic M Anderla A Culibrk D Stefanovic D 2016 Deep neural networks based recognition of plant diseases by leaf image classification Computational Intelligence and Neuroscience 2016 3289801 10.1155/2016/3289801 27418923 PMC4934169

[b134-tjb-49-05-459] Sood S Singh H 2023 A comparative study of grape crop disease classification using various transfer learning techniques Multimedia Tools and Applications 83 4359 4382 10.1007/s11042-023-14808-0

[b135-tjb-49-05-459] Sun J Yang Y He X Wu X 2020 Northern maize leaf blight detection under complex field environment based on deep learning IEEE Access 8 33679 33688 10.1109/ACCESS.2020.2973658

[b136-tjb-49-05-459] Sun Y Jiang Z Zhang L Dong W Rao Y 2019 Slic svm based leaf diseases saliency map extraction of tea plant Computers and Electronics in Agriculture 157 102 109 10.1016/j.compag.2018.12.042

[b137-tjb-49-05-459] Szegedy C Ioffe S Vanhoucke V Alemi AA 2017 Inception-v4, inception-resnet and the impact of residual connections on learning 31st AAAI Conference on Artificial Intelligence AAAI 2017 4278 4284

[b138-tjb-49-05-459] Szegedy C Liu W Jia Y Sermanet P Reed S 2015 Going deeper with convolutions Proceedings of the IEEE Conference on Computer Vision and Pattern Recognition (CVPR) 1 9

[b139-tjb-49-05-459] Tabbakh A Barpanda SS 2023 A deep features extraction model based on the transfer learning model and vision transformer “tlmvit” for plant disease classification IEEE Access 11 45377 45392 10.1109/ACCESS.2023.3273317

[b140-tjb-49-05-459] Talasila S Rawal K Sethi G Sanjay MSS Reddy M 2022 Black gram plant leaf disease (bpld) dataset for recognition and classification of diseases using computer-vision algorithms Data in Brief 45 108725 10.1016/j.dib.2022.108725 36426030 PMC9679724

[b141-tjb-49-05-459] Tandirovic Gursel A Kaya Y 2025 Mam-incept-net: a novel inception model for precise interpretation of mammography images PeerJ Computer Science 11e3149 10.7717/peerj-cs.3149 PMC12453803 40989348

[b142-tjb-49-05-459] Tembhurne JV Gajbhiye SM Gannarpwar VR Khandait HR Goydani PR 2023 Plant disease detection using deep learning based mobile application Multimedia Tools and Applications 82 27365 27390 10.1007/s11042-023-14541-8

[b143-tjb-49-05-459] Terentev A Dolzhenko V Fedotov A Eremenko D 2022 Current state of hyperspectral remote sensing for early plant disease detection: A review Sensors 22 10.3390/s22030757 PMC8839015 35161504

[b144-tjb-49-05-459] Thangaraj R Anandamurugan S Kaliappan VK 2021 Automated tomato leaf disease classification using transfer learning-based deep convolution neural network Journal of Plant Diseases and Protection 128 73 86 10.1007/s41348-020-00403-0

[b145-tjb-49-05-459] Thapa R Zhang K Snavely N Belongie S Khan A 2020 The plant pathology challenge 2020 data set to classify foliar disease of apples Applications in Plant Sciences 8e11390 10.1002/aps3.11390 PMC7526434 33014634

[b146-tjb-49-05-459] Topuz EK Kaya Y 2025 Eo-lgbm-har: A novel meta-heuristic hybrid model for human activity recognition Computers in Biology and Medicine 189 110004 10.1016/j.compbiomed.2025.110004 40101582

[b147-tjb-49-05-459] Trivedi VK Shukla PK Pandey A 2022 Automatic segmentation of plant leaves disease using min–max hue histogram and k-mean clustering Multimedia Tools and Applications 81 20201 20228 10.1007/s11042-022-12518-7

[b148-tjb-49-05-459] Uguz S Uysal N 2021 Classification of olive leaf diseases using deep convolutional neural networks Neural Computing and Applications 33 4133 4149 10.1007/s00521-020-05235-5

[b149-tjb-49-05-459] Vallabhajosyula S Sistla V Kolli VKK 2022 Transfer learning-based deep ensemble neural network for plant leaf disease detection Journal of Plant Diseases and Protection 129 545 558 10.1007/s41348-021-00465-8

[b150-tjb-49-05-459] Verma S Kumar P Singh JP 2023 A meta-learning framework for recommending cnn models for plant disease identification tasks Computers and Electronics in Agriculture 207 107708 10.1016/j.compag.2023.107708

[b151-tjb-49-05-459] Wang H Ding J He S Feng C Zhang C 2023 Mfbp-unet: A network for pear leaf disease segmentation in natural agricultural environments Plants 12 10.3390/plants12183209 PMC10537337 37765373

[b152-tjb-49-05-459] Wang S Xu D Liang H Bai Y Li X 2025 Advances in deep learning applications for plant disease and pest detection: A review Remote Sensing 17 10.3390/rs17040698

[b153-tjb-49-05-459] Wiesner-Hanks T Wu H Stewart E DeChant C Kaczmar N 2019 Millimeter level plant disease detection from aerial photographs via deep learning and crowdsourced data Frontiers in Plant Science 10 10.3389/fpls.2019.01550 PMC6927297 31921228

[b154-tjb-49-05-459] Wilton A 2019 New zealand fungal and plant disease collection (pdd)

[b155-tjb-49-05-459] Wu SG Bao FS Xu EY Wang YX Chang YF 2007 A leaf recognition algorithm for plant classification using probabilistic neural network 2007 IEEE International Symposium on Signal Processing and Information Technology 11 16 10.1109/ISSPIT.2007.4458016

[b156-tjb-49-05-459] Xia L Zhang R Chen L Li L Yi T 2021 Evaluation of deep learning segmentation models for detection of pine wilt disease in unmanned aerial vehicle images Remote Sensing 13 10.3390/rs13183594

[b157-tjb-49-05-459] Xu L Cao B Zhao F Ning S Xu P 2023 Wheat leaf disease identification based on deep learning algorithms Physiological and Molecular Plant Pathology 123 10 1940 10.1016/j.pmpp.2022.101940

[b158-tjb-49-05-459] Yalcin H Razavi S 2016 Plant classification using convolutional neural networks 2016 Fifth International Conference on Agro-Geoinformatics (Agro-Geoinformatics) 1 5 10.1109/Agro-Geoinformatics.2016.7577698

[b159-tjb-49-05-459] Yin ZB Liu FY Geng H Xi YJ Zeng DB 2024 A high-precision jujube disease spot detection based on ssd during the sorting process Plos one 19e0296314 10.1371/journal.pone.0296314 PMC10769016 38180957

[b160-tjb-49-05-459] Yong LZ Khairunniza-Bejo S Jahari M Muharam FM 2023 Automatic disease detection of basal stem rot using deep learning and hyperspectral imaging Agriculture 13 10.3390/agriculture13010069

[b161-tjb-49-05-459] Yue X Qi K Na X Liu Y Yang F 2025 Deep learning for recognition and detection of plant diseases and pests Neural Computing and Applications 37 11265 11310 10.1007/s00521-025-11125-5

[b162-tjb-49-05-459] Zhang S Zhang C 2023 Modified u-net for plant diseased leaf image segmentation Computers and Electronics in Agriculture 204 107511 10.1016/j.compag.2022.107511

[b163-tjb-49-05-459] Zia Ur Rehman M Ahmed F Attique Khan M Tariq U Shaukat Jamal S 2021 Classification of citrus plant diseases using deep transfer learning Computers, Materials & Continua 70 10.32604/cmc.2022.019046

